# A high spatial resolution synchrotron Mössbauer study of the Tazewell IIICD and Esquel pallasite meteorites

**DOI:** 10.1111/maps.12841

**Published:** 2017-03-15

**Authors:** Roberts Blukis, Rudolf Rüffer, Aleksandr I. Chumakov, Richard J. Harrison

**Affiliations:** ^1^ Department of Earth SciencesUniversity of CambridgeCambridgeCB2 3EQUK; ^2^ European Synchrotron Radiation FacilityCS 40220F‐38043GrenobleFrance

## Abstract

Metallic phases in the Tazewell IIICD iron and Esquel pallasite meteorites were examined using ^57^Fe synchrotron Mössbauer spectroscopy. Spatial resolution of ~10–20 μm was achieved, together with high throughput, enabling individual spectra to be recorded in less than 1 h. Spectra were recorded every 5–10 μm, allowing phase fractions and hyperfine parameters to be traced along transects of key microstructural features. The main focus of the study was the transitional region between kamacite and plessite, known as the “cloudy zone.” Results confirm the presence of tetrataenite and antitaenite in the cloudy zone as its only components. However, both phases were also found in plessite, indicating that antitaenite is not restricted exclusively to the cloudy zone, as previously thought. The confirmation of paramagnetic antitaenite as the matrix phase of the cloudy zone contrasts with recent observations of a ferromagnetic matrix phase using X‐ray photoemission electron spectroscopy. Possible explanations for the different results seen using these techniques are proposed.

## Introduction

Recently, there has been increased interest in the magnetism of meteoritic Fe‐Ni alloys (Uehara et al. 2011; Bryson et al. 2014a, 2014b, 2015; Dos Santos et al. 2015; Nichols et al. 2016). Meteoritic metal alloys have traditionally been assumed to be rather poor paleomagnetic recorders. This is because the magnetic signal of bulk measurements is dominated by kamacite, a soft multidomain ferromagnet. However, more advanced, higher resolution magnetic imaging methods, as well as chemical extraction of particular metal phases, have shown that there are hard ferromagnetic phases present that can potentially retain useful paleomagnetic signals (Bryson et al. 2014a; Dos Santos et al. 2015). This discovery has renewed interest in meteorite magnetism, with attention focusing most intensely on the nanoscale intergrowth known as the “cloudy zone” (CZ) (Reuter and Williams 1988; Yang et al. 1997a, 1997b; Bryson et al. 2014b).

The CZ forms by spinodal decomposition of metastable face‐centered cubic (fcc) Fe‐Ni alloy (taenite) with composition ~24–47 at% Ni. As most meteoritic metal has bulk composition <24 at% Ni, CZ only forms in regions where the Ni concentration of taenite has been enriched by nucleation and growth of nearby kamacite. Kamacite has a body‐centered cubic (bcc) structure, and does not accept more than ~5 at% Ni in its structure (Yang and Williams 1996). As kamacite lamellae grow during slow cooling on the meteorite parent body, Ni builds up ahead of the advancing kamacite/taenite interface, forming a Ni‐rich rim. Depending on the cooling rate and initial Fe‐Ni ratio, the Ni concentration can reach ~67 at% (Yang et al. 1997a, 1997b). However, in most Fe‐Ni meteorites, it reaches only ~50 at%. The Ni concentration is highest at the kamacite/taenite interface and decays approximately exponentially to the average bulk composition of the meteorite farther away from the interface (Yang and Goldstein 2005). At temperatures between ~100 and 450 °C, the high Ni areas beyond the rim unmix via a mechanism of spinodal decomposition into Ni‐rich (~50 at% Ni) islands and a Ni‐poor (~15 at% Ni) matrix. Spinodal decomposition begins at a higher temperature for higher Ni concentrations. The combination of longer diffusion distances at high temperature and the availability of more time for coarsening to take place during cooling, results in a coarser spinodal microstructure in more Ni‐rich regions. Consequently, islands are larger near the kamacite/taenite interface and decrease in size with increasing distance from the interface. Islands order below ~320 °C to form tetrataenite, a tetragonal form of FeNi with the L10 superstructure. Far from the interface, where the composition is <25 at% Ni, a low‐temperature martensitic transition is encountered. This transforms the low‐Ni taenite into bcc martensite, which subsequently decomposes into a two‐phase mixture of low‐Ni bcc and high Ni‐fcc phases, known as plessite (Goldstein and Michael 2006).

Due to its tetragonal structure, tetrataenite is a hard ferromagnet (Néel et al. 1964), and because of their small size, the tetrataenite islands in the CZ are assumed to be single domain (Uehara et al. 2011). This makes the CZ a potentially good paleomagnetic recorder. However, the recording properties of the CZ also depend on the magnetic properties of the matrix.

While tetrataenite has been relatively well studied (Néel et al. 1964; Albertsen 1981; Lima and Drago 2001; Bordeaux et al. 2016), the properties of the matrix phase are less well constrained. From mass balance and synchrotron studies (Scorzelli 2008), the matrix composition has been inferred to be in range of 63–87 at% Fe, depending on the method (Miller and Russell 1989; Rancourt et al. 1999; Scorzelli et al. 2007). The matrix has variously been proposed to be bcc kamacite, paramagnetic disordered taenite, ferromagnetic ordered Fe_3_Ni, and a low‐moment disordered form of taenite called antitaenite (Danon et al. 1979; Rancourt and Scorzelli 1995). Antitaenite has only been directly detected using Mössbauer spectroscopy of bulk meteorite samples (Rancourt et al. 1999). The precise location of antitaenite within the microstructure cannot be determined using bulk measurements, but it is thought to only exist in the CZ, as a fine‐scale intergrowth with tetrataenite (Rancourt et al. 1999; Scorzelli et al. 2007; Bryson et al. 2014b). The coherency strain associated with the intergrowth acts to stabilize antitaenite against the martensitic transition. Antitaenite is proposed to have a low‐moment electronic structure that is distinct from that of high‐moment fcc taenite, resulting in a characteristically low value of the isomer shift observed by Mössbauer spectroscopy (Rancourt et al. 1999).

Paleomagnetic information can be extracted from the CZ using X‐ray photoemission electron microscopy (XPEEM) (Bryson et al. 2014a, 2015). The highest resolution images obtained to date (Nichols et al. 2016) indicate that all components of CZ are magnetic. No nonmagnetic or low‐moment phases have been observed with this method. This observation is at odds with the prevailing view that matrix phase is paramagnetic antitaenite. To enable accurate paleomagnetic reconstruction, the properties of the matrix have to be known. Here we use synchrotron Mössbauer spectroscopy applied for the first time to meteorite samples, to obtain spatially resolved spectral information at 5–10 μm intervals across the kamacite–CZ–plessite transition. We use this method to look for evidence of a magnetic matrix phase. A bulk Mössbauer study of the CZ has been performed for the Santa Catharina ataxite (Rancourt et al. 1999), an unusually Ni‐rich meteorite consisting almost entirely of CZ. Although no magnetic matrix was found, Santa Catharina has a microstructure that is atypical of the group of meteorites currently being used for nanopaleomagnetic analysis. Our aim was to obtain spatially resolved Mössbauer spectra throughout the CZ of a more conventional Widmanstätten microstructural sequence.

## Experimental Methods

A sample of the Esquel pallasite (sample No. BM.1964,65) was obtained from the Natural History Museum London, UK. A small piece of the Tazewell meteorite (sample No. 16269) was provided by the Sedgwick Museum of Earth Sciences, University of Cambridge, UK. The same samples were previously used for transmission electron microscopy (TEM) and XPEEM analysis (Bryson et al. 2014a, 2014b, 2015). Samples were prepared by mechanical polishing on a carbide paper to the desired thickness of 20–40 μm. For examination in the SEM, the samples were further mechanically polished with water‐based monocrystalline diamond suspension, with the final particle diameter of 0.25 μm.

Scanning electron microscopy (SEM) images and electron backscatter diffraction (EBSD) and energy dispersive spectroscopy (EDS) data were obtained using FEI QUANTA 650F SEM in high vacuum. Backscattered electron (BSE) images were obtained using 10 kV acceleration voltage. EBSD patterns were obtained using 20 kV acceleration voltage, ~6 nA, working distance of 14.7 mm, sample detector distance of 16 mm, and sample tilt of 70° consisting of 45° of sample holder tilt and 25° of stage tilt. Bruker e^−^Flash^HR^ and Bruker XFlash 6130 detectors were used for EBSD and EDS, respectively.

Synchrotron Mössbauer spectroscopy was performed with the Synchrotron Mössbauer Source (SMS) (Rüffer and Chumakov 1996) at the Nuclear Resonance beamline ID18 at the European Synchrotron Radiation Facility in Grenoble, France (Smirnov et al. 1997). The beam from SMS is nearly 100% polarized with the electric vector in the vertical direction. Typical count rates on the sample reach 16 × 10^3^ resonant quanta per second. The beam shape is approximately Gaussian, and can be focused to a spot size of 9 × 4 μm^2^ FWHM under ideal conditions (Rüffer and Chumakov 1996). For our experiment, focused beam sizes [h × v] of 12 × 16.5 μm^2^ and 12 × 40 μm^2^ FWHM for the Esquel and Tazewell samples, respectively, were achieved. The energy distribution of the source follows a Lorentzian‐squared distribution with a linewidth of 0.21 mm s^−1^. The Doppler shift was provided by oscillating the ^57^FeBO_3_ nuclear monochromator with a sinusoidal drive and maximum velocity of 11.25 mm s^−1^ distributed over 512 velocity channels in the multichannel analyzer. All spectra were acquired at room temperature ~22 °C. The storage ring was operated in uniform filling mode.

Samples were mounted over holes in an Al holder. The holder was bolted to a large moving stage with ±1 μm accuracy. Sample navigation was achieved by first mapping changes in X‐ray absorption as the sample was rastered across the X‐ray beam, enabling the XY stage positions corresponding to prominent features around the sample edge to be determined. The locations of individual spectral measurements were then determined by triangulation of the stage position relative to SEM images of the samples on the holder. Using this method, we estimated the uncertainty in the absolute position of individual spectral measurements to be ~10–20 μm. The uncertainty in the relative position of spectral measurements within a single profile is equal to the accuracy of the stage movement.

Due to the high intensity of synchrotron radiation, average spectrum acquisition time was ~2800 s. In total, 56 spectra were obtained over 2.5 days, a rate of data acquisition that is not accessible with a conventional Mössbauer experiment using a radiogenic source.

The spectra were analyzed using the MossA software package using full transmission integral approach (Prescher et al. 2012). Errors for all Mössbauer data presented on graphs and in text are equal to twice the standard deviation as obtained from fitting statistics. The errors presented in the Mössbauer parameter data table are equal to a single standard deviation (see Table S1 in supporting information). The phases were treated as absorbers having Lorentzian absorption profiles while the emission profile of the synchrotron Mössbauer source was described by a Lorentzian‐squared function (Potapkin et al. 2012). Lorentzian line shape was chosen to describe the absorbers, as all Fe‐containing phases were assumed to be pure with a low degree of disorder that would introduce a Gaussian component. After obtaining the area under the curve for each phase, the phases were normalized based on their Fe content. It was assumed that there is no isotopic fractionation of ^57^Fe between the phases. The Fe contents for kamacite, antitaenite, tetrataenite, and schreibersite were assumed to be 95 at%, 85 at%, 50 at%, and 35 at%, respectively. The values for the first three phases were based on electron probe/TEM observations of the Esquel meteorite (Desrousseaux et al. 1997). The Fe content of schreibersite was estimated from EDS data obtained simultaneously with EBSD spectra. These phases were identified based on well‐known properties such as isomer shift, hyperfine splitting, and quadrupole splitting used previously by Oshtrakh et al. (2008) and Dos Santos et al. (2015).

## Results

### Mössbauer Spectroscopy

**Figure 1:**
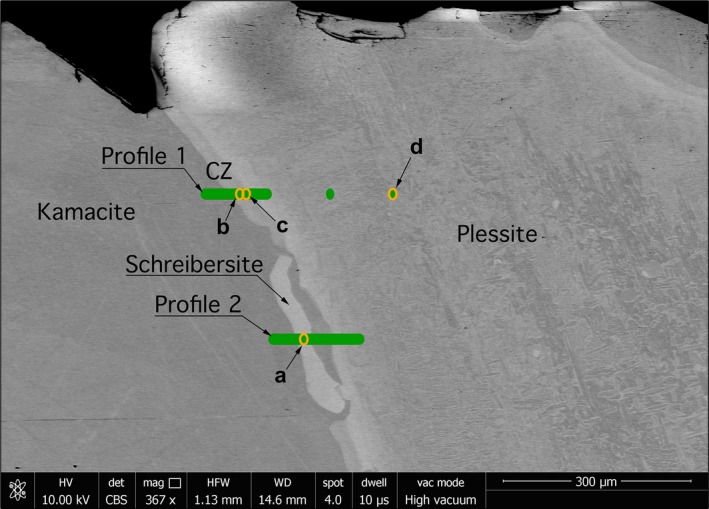
Scanning electron microscopy (SEM) image in backscatter mode (BSE) of the Esquel meteorite sample. Green bars mark areas where spectra were obtained, forming two horizontal line profiles. Profiles start at the left‐hand side. Orange ovals mark sites where spectra shown in Fig. 2 were taken. The size of the ovals represents the size of the beam. [Color figure can be viewed at wileyonlinelibrary.com]

**Figure 2:**
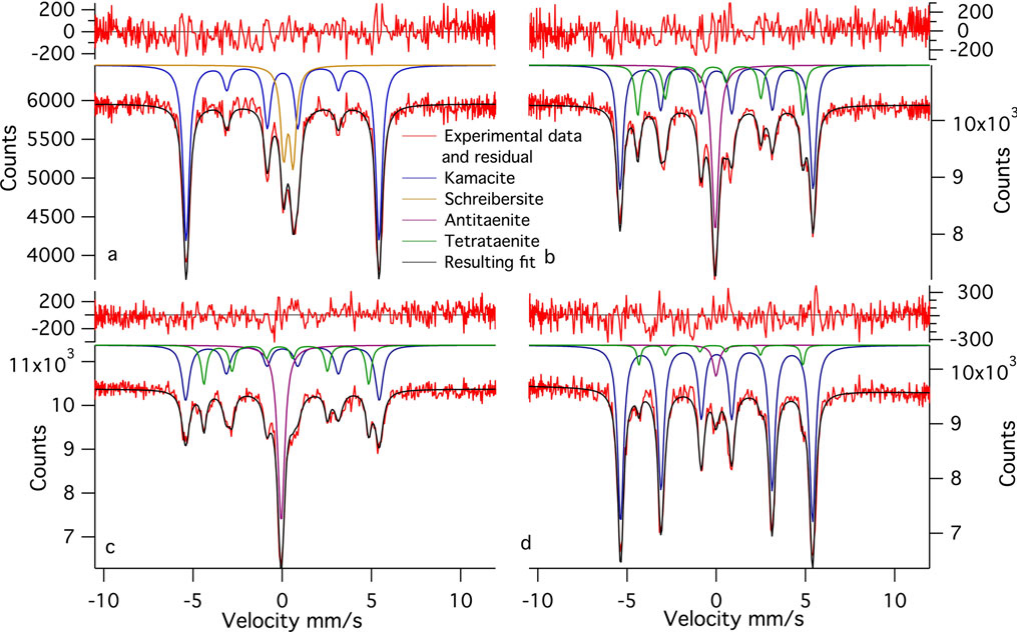
A selection of representative Mössbauer spectra from the Esquel meteorite: a) spectrum containing schreibersite; b) spectrum acquired in the coarse cloudy zone, closer to the large kamacite lamella; c) spectrum acquired in the fine cloudy zone, closer to plessite; and d) spectrum acquired deep into plessite. [Color figure can be viewed at wileyonlinelibrary.com]

**Figure 3:**
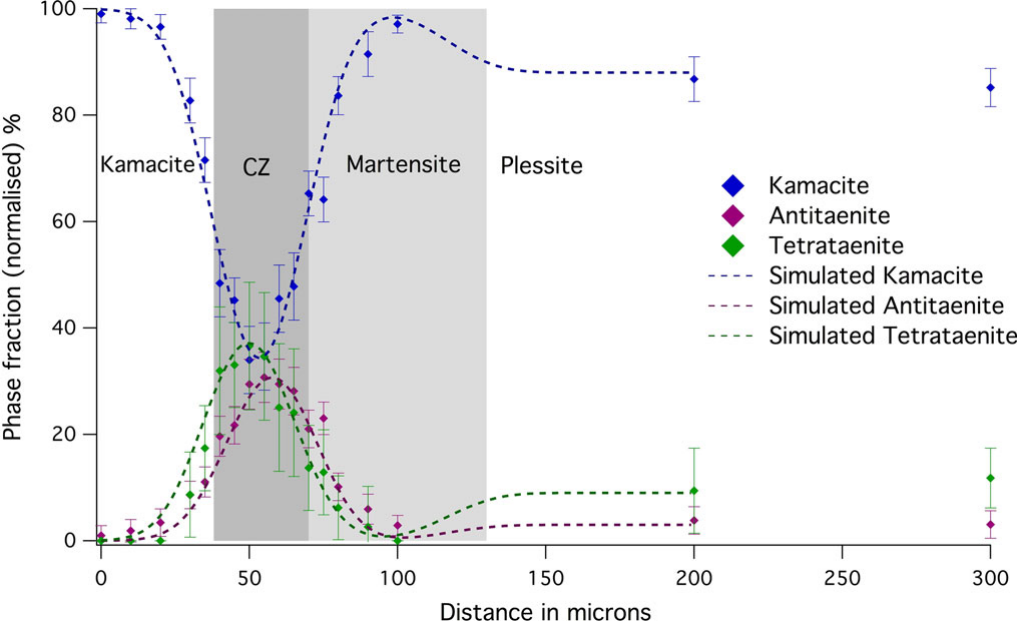
Variation in the abundance of phases throughout the cloudy zone, from kamacite to plessite, as determined from Mössbauer spectra taken along Profile 1 of the Esquel meteorite (Fig. 1). [Color figure can be viewed at wileyonlinelibrary.com]

Two line profiles were obtained from the Esquel meteorite sample (Fig. 1). The first‐line profile transects the kamacite–CZ–plessite transition. Both single‐phase martensite and multiphase plessite were identified in BSE images (Fig. 1). Mössbauer spectra were obtained every 5 μm while crossing the CZ. Overall 18 spectra along a 300 μm long section were taken. Only three phases were reliably detected: kamacite, antitaenite, and tetrataenite (Fig. 2). A decrease in kamacite content is observed as the kamacite/taenite interface is crossed, accompanied by a corresponding increase in tetrataenite and antitaenite (Fig. 3). The peak in antitaenite phase fraction occurs some 5–10 μm farther from the kamacite/taenite interface than the tetrataenite peak. After passing the CZ, the amount of kamacite rises to a local maximum at 100 μm from the kamacite/taenite interface, and there is a corresponding local minimum in tetrataenite. Beyond 100 μm, kamacite decreases and tetrataenite increases, whereas antitaenite remains constant throughout the plessite region. The sum of the two fcc phases tetrataenite and antitaenite make up 14.9 at% of total area in plessite.

The magnetic hyperfine field of kamacite varies throughout the profile. The hyperfine field in the kamacite lamella is 33.58 ± 0.08 T. This increases significantly as plessite is approached and peaks at 33.75 ± 0.14 in the martensite region between the CZ and plessite. The hyperfine field is much lower (33.39 ± 0.06 T) in plessite. There are systematic variations in the linewidth of kamacite that can be explained as overlap of two types of kamacite with slightly different hyperfine fields (see supporting information).

**Figure 4:**
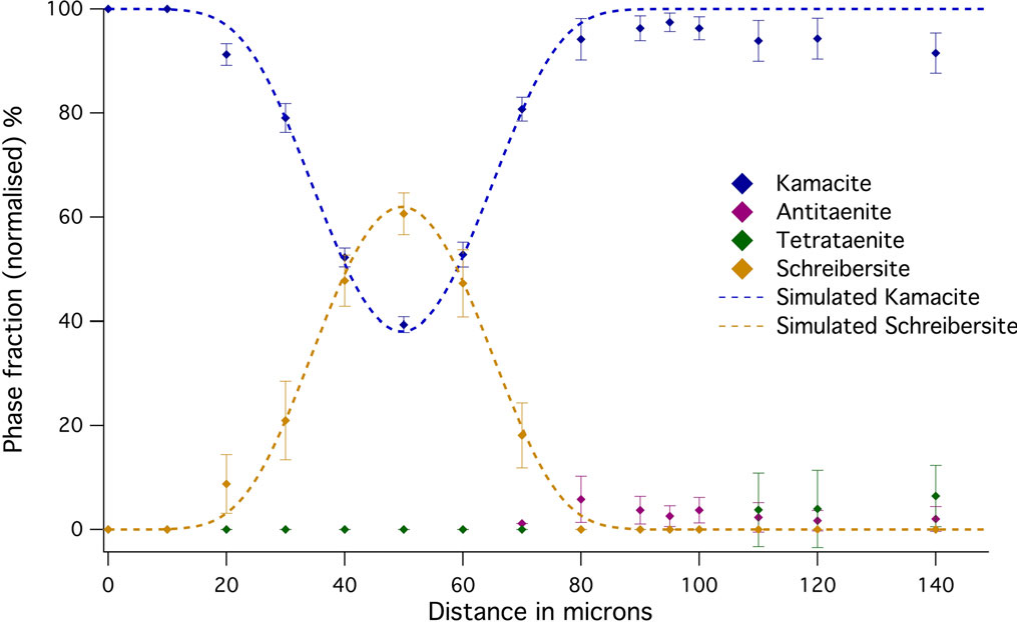
Variation in the abundance of phases along a traverse of a schreibersite grain, from kamacite to plessite, as determined from Mössbauer spectra taken along Profile 2 of the Esquel meteorite (Fig. 1). Deviations between the observed and modeled proportions on the right‐hand side are due to the approximation of plessite as pure kamacite in the model. [Color figure can be viewed at wileyonlinelibrary.com]

A profile consisting of 15 spectra over 140 μm was made across a grain of schreibersite, close to the kamacite/taenite interface (Fig. 4). The profile starts in kamacite and ends in plessite. This profile does not contain tetrataenite, except for small amounts in plessite. No CZ was observed. Small amounts of fcc material were observed using EBSD (see the following section); however, the width was only 0–3 μm, and therefore it was potentially too little to be reliably detected by Mössbauer spectroscopy. Even though the width of the phosphide grain was ~30 μm, which is larger than the beam size used, significant amounts of kamacite are present in all spectra. This is most likely due to oblique angle at which the grain intersects the surface as well as the beam tails that contribute to the spectra.

Hyperfine fields and isomer shifts of kamacite are constant, within error (Table 1).

**Table 1:** Energy dispersive spectroscopy (EDS) data obtained simultaneously with EBSD data. Errors presented are equal to a single standard deviation

Data set	Phase	Fe at%	Ni at%	P at%
Profile 1 plessite region	Plessite average	88 ± 2	12.5 ± 0.4	–
Profile 1 plessite region	Plessite kamacite	96 ± 3	3.85 ± 0.14	–
Profile 1 near CZ	Kamacite lamella	94 ± 3	5.83 ± 0.19	–
Profile 1 near CZ	Martensite within 5 μm of CZ	78 ± 2	22.4 ± 0.7	–
Profile 1 near CZ	CZ within 5 μm of martensite	76 ± 2	23.8 ± 0.7	–
Profile 1 near CZ	Martensite within 5 μm of plessite	84 ± 2	16.0 ± 0.5	–
Profile 1 near CZ	Tetrataenite rim	56 ± 1	44.3 ± 1.3	–
Profile 2	Kamacite near schreibersite	94 ± 3	5.8 ± 0.2	–
Profile 2	Kamacite between schreibersite and plessite	96 ± 3	4.44 ± 0.16	–
Profile 2	Thin fcc area near schreibersite	60 ± 2	40.0 ± 1.2	–
Profile 2	Martensite	79 ± 2	20.9 ± 0.7	–
Profile 2	Schreibersite	36 ± 1	41.7 ± 1.3	22.4 ± 0.5

**Figure 5:**
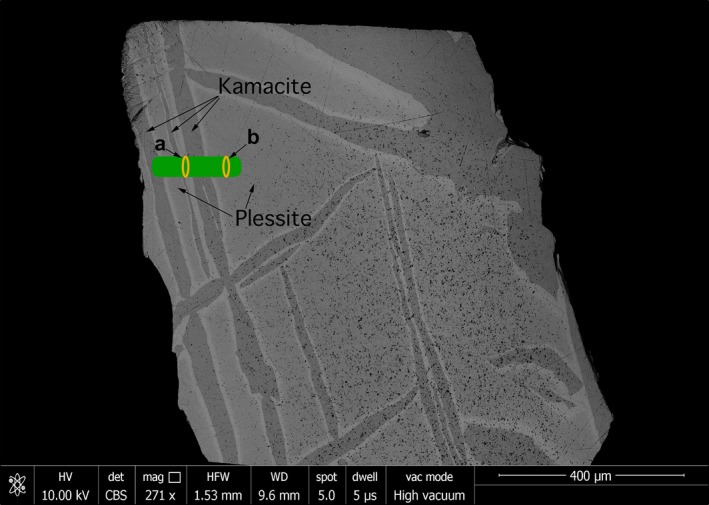
Scanning electron microscopy (SEM) backscattered electron (BSE) image of the Tazewell meteorite. Green bar marks an area where spectra were obtained forming a horizontal line profile. Profile starts at the left‐hand side. Orange ovals mark areas where spectra shown in Fig. S8 in supporting information were taken. The size of the ovals corresponds to the size of the beam. [Color figure can be viewed at wileyonlinelibrary.com]

**Figure 6:**
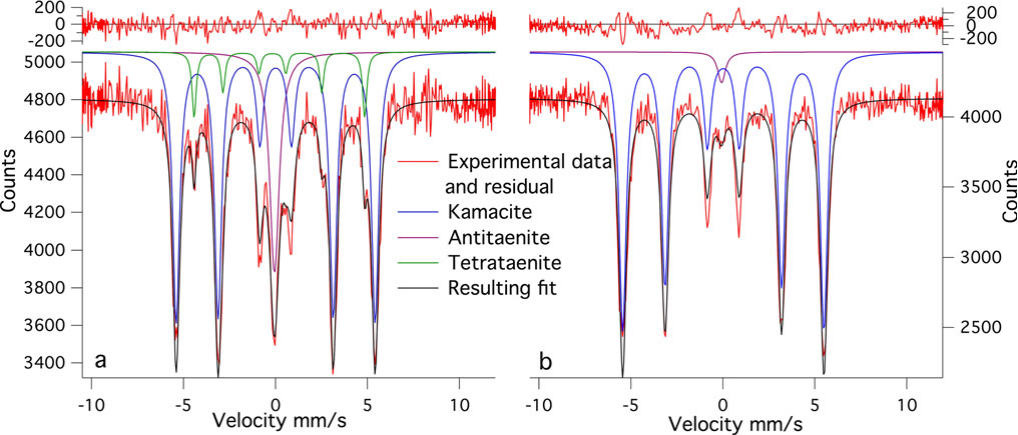
Two representative Mössbauer spectra from the Tazewell meteorite showing: a) cloudy zone and b) plessite. [Color figure can be viewed at wileyonlinelibrary.com]

**Figure 7:**
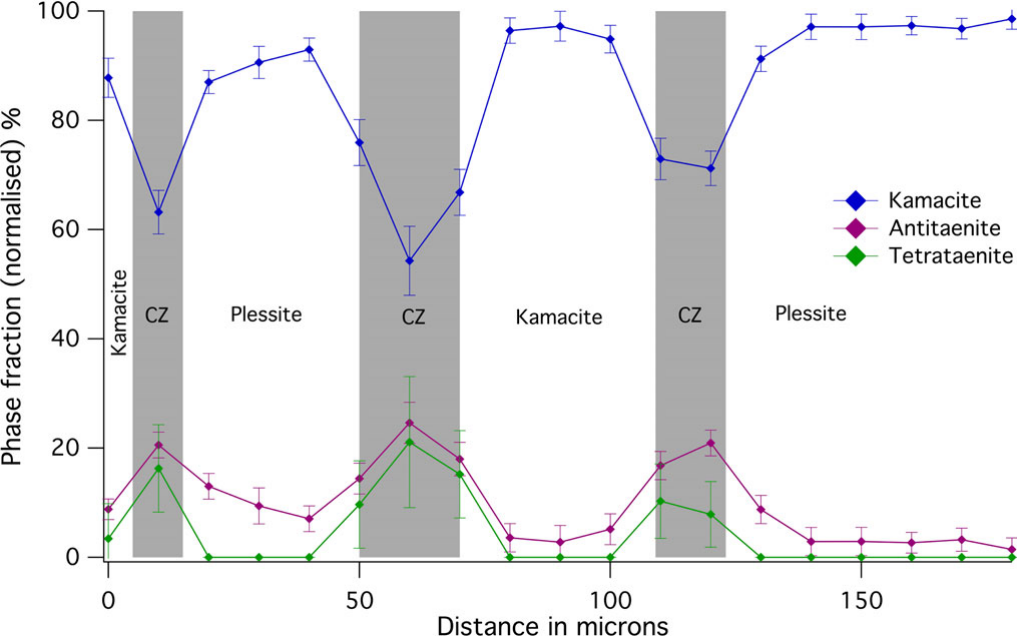
Variations in the abundance of phases obtained from the Tazewell meteorite. The broader beam and finer metallurgical features cause kamacite to be the most highly abundant phase in this sample. [Color figure can be viewed at wileyonlinelibrary.com]

A 180 μm long line profile, with spectra collected every 10 μm was obtained for the Tazewell IIICD meteorite (Figs. 5 and 6). The profile begins in a kamacite lamella, crosses a 40 μm wide plessite region followed by a second 40 μm wide kamacite lamella, before terminating in a large plessite region (Fig. 7). Due to the smaller scale of the metallurgical features in the Tazewell meteorite compared to the Esquel, some of the fine details observed in Fig. 2 are lost. The broader vertical focus of the beam (12 × 40 μm instead of 12 × 16.5 μm) also contributes to this. The presence of CZ can be clearly seen as an increase in tetrataenite and antitaenite fraction relative to kamacite. An interesting feature is the high proportion of antitaenite and lack of tetrataenite observed while crossing the first plessite region. There are small amounts of antitaenite detected in kamacite lamellae. This is likely to be an artifact caused by the contribution of beam tails sampling nearby CZ. In addition, antitaenite contains more Fe than tetrataenite and is therefore easier to detect. We estimate the detection limit for Fe in tetrataenite as 3 at%, corresponding to an error of ~±6 phase % tetrataenite. The errors of phase % shown in the graphs were obtained from fitting parameters and therefore are zero if the phase is not present, i.e., was not fitted. It is possible the phase is present but at lower amounts than the detection limit. Plessite in the Tazewell has a rather different composition to that observed in Esquel: Esquel plessite contains significant quantities of tetrataenite and small amounts of antitaenite; Tazewell plessite contains no measurable tetrataenite and low levels of antitaenite.

Magnetic hyperfine fields of kamacite in the Tazewell show rather different trends to those observed in Esquel, with kamacite lamellae showing lower hyperfine fields in the lamella than in plessite. However, the magnitude of hyperfine field in kamacite lamellae are the same, within error, in both samples (~33.6 T). Hyperfine field in plessite of Tazewell sample shows similar values to martensite in Esquel sample. Changes in isomer shift are observed in the profile. The isomer shift is lower in plessite than in kamacite. However, the changes in isomer shift are of comparable magnitude to the error; therefore, it cannot be conclusively determined how significant these changes are. This difference in isomer shift was not observed in the Esquel sample.

The center shift of kamacite was found to be 0.018 ± 0.013 mm s^−1^, which was the same in both samples. For antitaenite, a center shift values of −0.060 ± 0.012 mm s^−1^ in Esquel and −0.058 ± 0.014 mm s^−1^ in Tazewell samples were found, which, when corrected for second‐order Doppler effect, corresponds to 0.159 ± 0.012 mm s^−1^ and 0.161 ± 0.014 mm s^−1^, respectively. This is consistent with antitaenite being a low‐moment phase (Rancourt et al. 1999; Lagarec et al. 2001). For tetrataenite, the center shift was found to be 0.029 ± 0.005 mm s^−1^ in both samples. The isomer shifts reported for metal alloy phases were corrected for second‐order Doppler shift using the Debye approximation, as described in Nasu and Murakami (1971). The Debye temperatures used were those given by Rancourt et al. (1999), which corresponds to −0.219 mm s^−1^ for fcc phases (Rancourt et al. 1992) relative to reference α‐Fe isomer shift at room temperature.

Fitting parameters of antitaenite and tetrataenite were examined, but not analyzed for linewidth, isomer shift changes, or other parameters, as the concentration of these phases was often very low, and therefore the fitting parameters have large uncertainties. In some cases, where a phase was clearly present but at a very low concentration (<5–10%), fitting parameters were constrained to those calculated as averages from reliable fits. If this was not done, the best mathematical fits were obtained by parameters that are not physically plausible.

### EBSD

EBSD was performed on Esquel at the same sites where Mössbauer line profiles were obtained (Figs. S1–3 in supporting information). The symmetry and lattice parameters of the phosphide grain are consistent with schreibersite (Fe,Ni)_3_P. As expected, the CZ is fcc. Kamacite lamellae are bcc. Plessite consists of differently oriented kamacite laths. Plessite also contains small thin rims of fcc material around kamacite lamellae, presumed to be tetrataenite as tetrataenite was observed in Mössbauer spectra of plessite. Deeper into plessite, 17.5% of the area consists of fcc material. In plessite, the {111} planes of the fcc phase coincides with {110} planes of bcc material, consistent with other authors (Goldstein and Michael 2006).

No significant fcc material in plessite was observed in the first 30–60 μm from the CZ, even though differently oriented lamellae are visible in orientation maps and BSE images due to surface relief caused by etching during polishing (see supporting information). Almost all significant fcc material in plessite has the same crystallographic orientation as the CZ with only a few small grains having a different one, in agreement with previous studies of plessite (Goldstein and Michael 2006). There are different areas of plessite marked by kamacite grain size and shape changes. Very small amounts of fcc material were observed near the schreibersite grain compared to the other examined region.

## Discussion

### Phase Fractions through the Esquel CZ

The quantitative analysis of Mössbauer spectra across the CZ in Esquel shows clearly that the peak in tetrataenite phase fraction occurs 5–10 μm ahead of the peak in antitaenite phase fraction (Fig. 3). The origin of this effect can be explained using a simple model of the transitional taenite region. The Ni concentration of the taenite region is approximated as an exponential function with a decay constant of 0.045, starting at 50% Ni at the tetrataenite rim; unit of length used in the model is μm. The Ni concentration decay was observed to fit an exponential function in electron microprobe measurements of the Tazewell meteorite. For Ni concentrations in the range 50–21 at%, the system is treated as CZ: a two‐phase mixture of tetrataenite and antitaenite, with varying phase proportions given by the lever rule. The compositions of tetrataenite and antitaenite are assumed to remain constant throughout the CZ (50 at% Ni and 15 at% Ni, respectively). This creates a CZ that is 25 μm wide, with an increasing proportion of antitaenite relative to tetrataenite with increasing distance from the kamacite/taenite interface. When Ni concentration falls below 21 at% Ni, the system is treated as martensite: a single‐phase band of Ni‐rich kamacite (~70 μm wide). The region beyond martensite is treated as plessite: a three‐phase mixture of kamacite, tetrataenite, and antitaenite, with phase proportions equal to those determined by Mössbauer spectroscopy far from the kamacite/taenite interface. The compositions of the CZ/martensite and martensite/plessite boundaries were selected based on EDS data, and are consistent with other work on this meteorite (Desrousseaux et al. 1997). The orientation of the interface was assumed to be rotated by 35° from vertical, relative to horizontal scanning direction shown in Fig. 1. The beam was approximated as a 2D Gaussian function with horizontal FWHM fixed at 12 μm and vertical FWHM fixed at 16.5 μm, as measured during the experiment. The samples were assumed to be 20 μm thick. The only remaining unknown variable is the inclination of the kamacite/taenite and CZ/martensite interfaces to the sample surface. This was manually adjusted during the calculation to optimize the agreement between the observations and the model, which reproduces both the delayed antitaenite peak in the fine CZ and the local minimum in tetrataenite within martensite. The model demonstrates that the ubiquitous presence of kamacite throughout the CZ is explained by the combination of beam overlap with kamacite and martensite regions, rather than presence of kamacite within the CZ itself.

### Magnetic Properties of the Matrix

The magnetic properties of the matrix phase are critical in determining the overall magnetic properties of the CZ, and thereby how the CZ acquires paleomagnetic remanence on cooling. The observed variations in phase fractions throughout the CZ are consistent with a model of ferromagnetic tetrataenite islands in a matrix of paramagnetic antitaenite. Previous Mössbauer studies of meteoritic Fe‐Ni alloy have observed a disordered, ferromagnetic, fcc taenite phase in addition to tetrataenite (Dos Santos et al. 2015). The presence of this phase was mostly explained as due to disordering of tetrataenite due to shock caused by impact events. Both meteorites used in this study are classed as low shock; therefore, a lack of disordered taenite is not surprising. Crucially, no evidence of distinct ferromagnetic matrix phase was found.

The absence of a ferromagnetic matrix phase contrasts starkly with the conclusions of XPEEM (Bryson et al. 2014a; Nichols et al. 2016) and electron holography (Bryson et al. 2014b) imaging studies of the CZ, which all point toward the presence of soft ferromagnetic matrix. It has also been argued on the basis of micromagnetic simulations that a soft magnetic matrix is necessary to explain the low coercivity of the coarse CZ compared to theoretical predictions of the coercivity of single‐domain tetrataenite (Bryson et al. 2014b). There are several possible ways to reconcile these observations, which we explore below.

Arguably the simplest possibility is that neither XPEEM nor electron holography have the necessary spatial resolution to reveal the true paramagnetic nature of the matrix phase. Although the spatial resolution of electron holography is, in principle, more than good enough to resolve features several tens of nm in size (Dunin‐Borkowski et al. 2011), it suffers from the fact that the quantity imaged is magnetic induction, B, rather than magnetization, M. This means the magnetic signal of the matrix is obscured by the stray magnetic field emanating from adjacent islands. Based on a qualitative comparison of observed and simulated electron holography images (together with several other lines of evidence), Bryson et al. (2014b) concluded that the matrix phase was likely to be a soft ferromagnet. However, given the presence of strong stray fields, such qualitative comparisons may not be 100% reliable. More convincing is evidence from XPEEM, which images M directly, and therefore does not suffer from the stray field issue. Nichols et al. (2016) provided evidence of a magnetic matrix phase from high‐resolution XPEEM images of Marjalahti pallasite. However, image simulations that include a paramagnetic matrix were not made for comparison, and even under ideal conditions, the maximum spatial resolution of XPEEM imaging is 30 nm—only just good enough to resolve an isolated patch of paramagnetic matrix in the coarse CZ. The instrumental broadening function used to simulate experimental XPEEM images is typically >30 nm, so it is conceivable that the paramagnetic matrix has not been fully resolved. A counterargument to this follows from XPEEM observations of fine‐scale CZ. As the Mössbauer spectroscopy data show, the amount of antitaenite increases relative to tetrataenite toward the fine CZ. Therefore, we would expect the total magnetic signal seen by XPEEM to be significantly lower in the fine CZ. XPEEM data, however, show that the magnetic signal remains strong throughout the CZ, and often increases significantly in the fine CZ. Therefore, it remains difficult to reconcile the XPEEM observations with Mössbauer observations based on spatial resolution arguments alone.

A second consideration is the rather different bulk versus surface sensitivities of the experimental techniques. Although synchrotron Mössbauer is performed on a thin slice (20–30 μm), it can be considered a “bulk” measurement in comparison to XPEEM, which only probes the upper ~5 nm of the sample surface, or electron holography, which is performed on a TEM foil typically <100 nm thick. It has been proposed that antitaenite is stabilized against martensitic transformation by its epitaxial contact with tetrataenite within the CZ (Scorzelli and Danon 1985; Chadwick 1997). Tetrataenite is tetragonal, and its lattice parameters differ slightly from cubic fcc lattice parameters c/a = 1.0036 (Albertsen 1981). This could induce stress that stabilizes antitaenite in the matrix (Scorzelli and Danon 1985). However, in thin films, such as TEM lamellae, or at the surface, this stress could be relaxed, allowing a different electronic and/or magnetic structure to form. Examples of metallic alloys that have very different electronic and magnetic structures in bulk versus surface measurements are documented (Schneider et al. 2006; Pressacco et al. 2016). It has also been shown that magnetization is enhanced at the surface relative to bulk in 3d metals (Punkkinen et al. 2011).

Third, it is conceivable that different sample preparation techniques (mechanical polishing, Ar‐ion sputtering, and focused Ga‐ion beam thinning for Mössbauer spectroscopy, XPEEM, and electron holography, respectively) create a change in the structural and/or magnetic properties of the matrix. It is well known that mechanical polishing induces a structural and magnetic change in the upper ~100 nm of the sample surface (Bryson et al. 2014a). This altered, soft magnetic layer is removed by Ar‐ion sputtering under high vacuum prior to XPEEM, but will be still present in the Mössbauer samples (albeit making up only a tiny fraction of the total thickness of the sample). Failure to remove the soft magnetic layer in XPEEM studies is considered unlikely, given that its presence leads to easily recognizable magnetic structures that differ considerably from those normally seen in the CZ. It is currently not known what, if any, effect Ar‐ion sputtering or focused ion beam thinning has on the matrix phase, and therefore it remains (an admittedly speculative) possible that sample preparation in some way contributes to the contrasting observations seen in Mössbauer and XPEEM.

Finally, micromagnetic simulations fail to reproduce the observed coercivity of coarse CZ without the presence of a soft ferromagnetic matrix that is exchange coupled to the tetrataenite islands (Bryson et al. 2014b). This soft matrix acts to dramatically lower the magnetic field required to nucleate the domain walls that enable the hard tetrataenite islands to reverse their magnetization. A possible way around the requirement for a ferromagnetic matrix is to invoke a concept originally put forward by Rancourt and Scorzelli (1997): the epitaxial contact that stabilizes antitaenite also allows a strong exchange contact between the ferromagnetic tetrataenite phase and the antitaenite phase. This interphase exchange coupling causes a partial ferromagnetic polarization of the antitaenite phase that penetrates a short distance (perhaps just a few atomic layers) into the matrix. Such a soft “ferromagnetic” shell, even if only a few nm in thickness, could significantly reduce the nucleation field for domains, in the same way as proposed for the ferromagnetic matrix by Bryson et al. (2014b). This hypothesis could be tested with micromagnetic simulation, but lies outside the scope of this experimental study.

### Martensite and Plessite

EBSD observations show the absence of an fcc phase in the region 30–70 μm from the CZ, referred to as martensite by some authors (Petrova et al. 2008). The Ni content of the martensite region is higher than kamacite lamellae (~20 at% versus 6 at%), and the hyperfine field is correspondingly higher (33.7 versus 33.3 T). Kamacite was found to have a hyperfine field value higher than pure synthetic α‐Fe (33 T). The difference is most simply explained by the ~5 at% Ni content of kamacite, which is known to increase hyperfine fields of α‐Fe up to ~20 at% Ni (Vincze et al. 1974). The increased Ni content in this region lowers the martensitic start temperature (*M*_s_) (Yang and Williams 1996) to the point where Ni cannot diffuse out of the growing kamacite laths. Plessite forms in Ni‐poor areas further from the kamacite/taenite interface, where *M*_s_ occurs at a higher temperature. As kamacite lenses, rods, and lamellae grow, Ni diffuses into the relict fcc taenite, forming Ni‐rich rims. These rims are mostly tetrataenite, but can also be less rich in Ni. Antitaenite is detected in low amounts within plessite in Tazewell and very likely in Esquel, implying either small amounts of CZ or pure antitaenite might be present, either as rims between kamacite grains or as secondary precipitates within tetrataenite rims. In Tazewell, due to its faster cooling rate of 10 K/10^6^y versus 1 K/10^6^y (Yang et al. 1997a, 1997b), Ni in plessite has not diffused fully into fcc rims. This leaves more Ni in kamacite leading to higher hyperfine fields than in Esquel plessite. The rims are left less Ni rich and therefore contain more antitaenite relative to tetrataenite than in Esquel. The rims around kamacite in plessite in Tazewell sample could therefore be mostly composed of either CZ or antitaenite.

### Schreibersite

The profile across schreibersite was simulated using a similar approach to that used for the Esquel CZ. It was assumed that the only two phases present are schreibersite and kamacite; hence, there are slight deviations in the plessite region. The aim of this simulation was to explain the experimental results and find the orientation of the grain. A set of conditions was found to simulate, within error, the observed pattern. The grain was rotated by 13° from vertical relative to horizontal scanning direction as observed in SEM image. In the same image, the grain was observed to vary in width near scanning position from 30 to 20 μm; for this simulation, the lower value of 20 μm was used. This is partially supported by qualitative observations of the reverse side of the sample, where the thickness and shape of the schreibersite grain differs only slightly. The beam size used and the sample thickness were the same as for the Esquel CZ. The inclination of the grain was found to be 56° relative to the surface. Therefore, the presence of kamacite in all spectra is caused by steep inclination of the featured being examined. The very small amounts of fcc phase observed quantitatively in Mössbauer spectra and qualitatively in BSE images are likely due to the low abundance of Ni as most of Ni has diffused into schreibersite, which contains 42 at% Ni based on EDS data (Table 1).

## Conclusions

Two meteorite samples were examined with synchrotron Mössbauer spectroscopy with high spatial resolution (~20 μm). Association of antitaenite with tetrataenite in the CZ was confirmed. No other phases were reliably detected in the CZ. However, antitaenite is not exclusively associated with the CZ as small amounts of it were also found in plessite. The hyperfine field of kamacite was found to vary in a predictable pattern if the diffusion of nickel at the time of kamacite growth is considered. A zone of martensite that has not exsolved to form plessite containing only bcc kamacite was indirectly detected with Mössbauer spectroscopy and directly imaged and detected with EBSD. As the matrix was found to be nonmagnetic, there remains an unresolved issue of discrepancy between Mössbauer spectroscopy measurements and methods such as XPEEM and electron holography. A proposed explanation is relaxation of the antitaenite to a ferromagnetic state once the strain imposed by tetrataenite islands is removed at the surface or in a thin film (TEM lamellae). The use of the CZ to extract paleomagnetic data by Bryson et al. (2015) was based on a simple thermodynamic argument, linking the biased populations of the six possible magnetic states of tetrataenite islands to the applied magnetic field and the island volume. These calculations did not consider explicitly the magnetic state of the matrix phase, although the presence of a magnetic matrix phase was included in the image simulations in order to better reproduce the experimental XPEEM images. Our results do not invalidate the conclusions of Bryson et al. (2015) in terms of the relative paleointensities extracted from the CZ, but they may well have some impact on attempts to put the results on an absolute paleointensity scale. Our results also serve to highlight the many fundamental questions that remain to be answered about the CZ remanence acquisition process. What is the magnetic state of individual taenite islands when they first form? What are their blocking temperatures and volumes? Is remanence blocked at temperatures above 320 °C, and if so, how is the remanence changed when the islands order to form tetrataenite below 320 °C? What is the influence of magnetostatic interactions between islands? The present study provides some clarity regarding the initial magnetic state of the system, which can now be modeled with some confidence as an interacting ensemble of individual taenite particles above 320 °C, and an interacting ensemble of individual tetrataenite particles below 320 °C. The initial acquisition of remanence by the CZ may be much closer to a conventional thermoremanent magnetization than previously thought, facilitating future attempts to model the process using existing theories. Micromagnetic modeling of interacting systems is now becoming routine (e.g., Einsle et al. 2016; Harrison and Lascu 2014), and will no doubt provide answers to these questions in time. Until then, it is recommended that interpretations of magnetic signals within the CZ are restricted to relative paleointensities only. Further experimental work to confirm the hypothesis of differing bulk versus surface magnetic properties is now required.

## Editorial Handling

Dr. A. J. Timothy Jull

## Supporting information


**Fig. S1**: Variations in hyperfine fields of kamacite throughout the cloudy zone, from Mössbauer spectra acquired along Profile 1 of the Esquel meteorite.

**Fig. S2**: Variations in linewidth (FWHM) of kamacite throughout the cloudy zone, from kamacite to plessite, as determined from Mössbauer spectra acquired along Profile 1 of the Esquel meteorite.

**Fig. S3**: Variations in the hyperfine field of kamacite across the profile of the Tazewell meteorite.

**Fig. S4**: Overlay of phases observed by EBSD over a BSE image of the Esquel meteorite at a site where Profile 1 was taken. Bcc iron is shown in red; fcc Fe‐Ni phases are green.

**Fig. S5**: Overlay of phases observed by EBSD over a BSE image of the Esquel meteorite at a site where Profile 2 was taken. Bcc Fe (kamacite) is shown in red; fcc Fe‐Ni phases are green; schreibersite is blue. Some misalignment of images, due to beam drift, can be seen at the top of the EBSD image.

**Fig. S6**: Overlay of phases observed by EBSD over a BSE image of the Esquel meteorite in plessite at the approximate location of the last spectrum in Profile 1. Bcc iron is shown in red; fcc Fe‐Ni phases are green.

**Fig. S7**: Pole figures of bcc Fe (left) and fcc Fe‐Ni (right) as obtained from plessite in the Esquel sample.

**Fig. S8**: EBSD images of the area shown in Fig. S4 showing: a) phases observed; bcc is red, fcc is green. b) Euler angles of bcc grains. c) Euler angles of fcc grains (CZ and rims in plessite). For color explanation of Euler angles see legend in Fig. S10.

**Fig. S9**: EBSD images of the area shown in Fig. S5 showing: a) phases observed; bcc is red, fcc is green, schreibersite is blue. b) Euler angles of fcc grains. c) Euler angles of bcc grains. d) Euler angle of schreibersite grain. Small single‐pixel sized, randomly oriented schreibersite grains are an artifact providing a qualitative indication of the number of misidentified and wrongly assigned pixels. For color explanation of Euler angles see legend in Fig. S10.

**Fig. S10**: Legend for Euler plots. Color of a pixel is a combination from contributions from all three Euler angles.

**Table S1**: Mössbauer fitting parameters for all spectra acquired.
