# Supplementary figures and images for: A high spatial resolution synchrotron Mössbauer study of the Tazewell IIICD and Esquel pallasite meteorites
Source: Meteorit Planet Sci. 2017 Mar 15;52(5):925–36. doi: 10.1111/maps.12841 (PMC5488627; doi:10.1111/maps.12841)

Kamacite B field, T

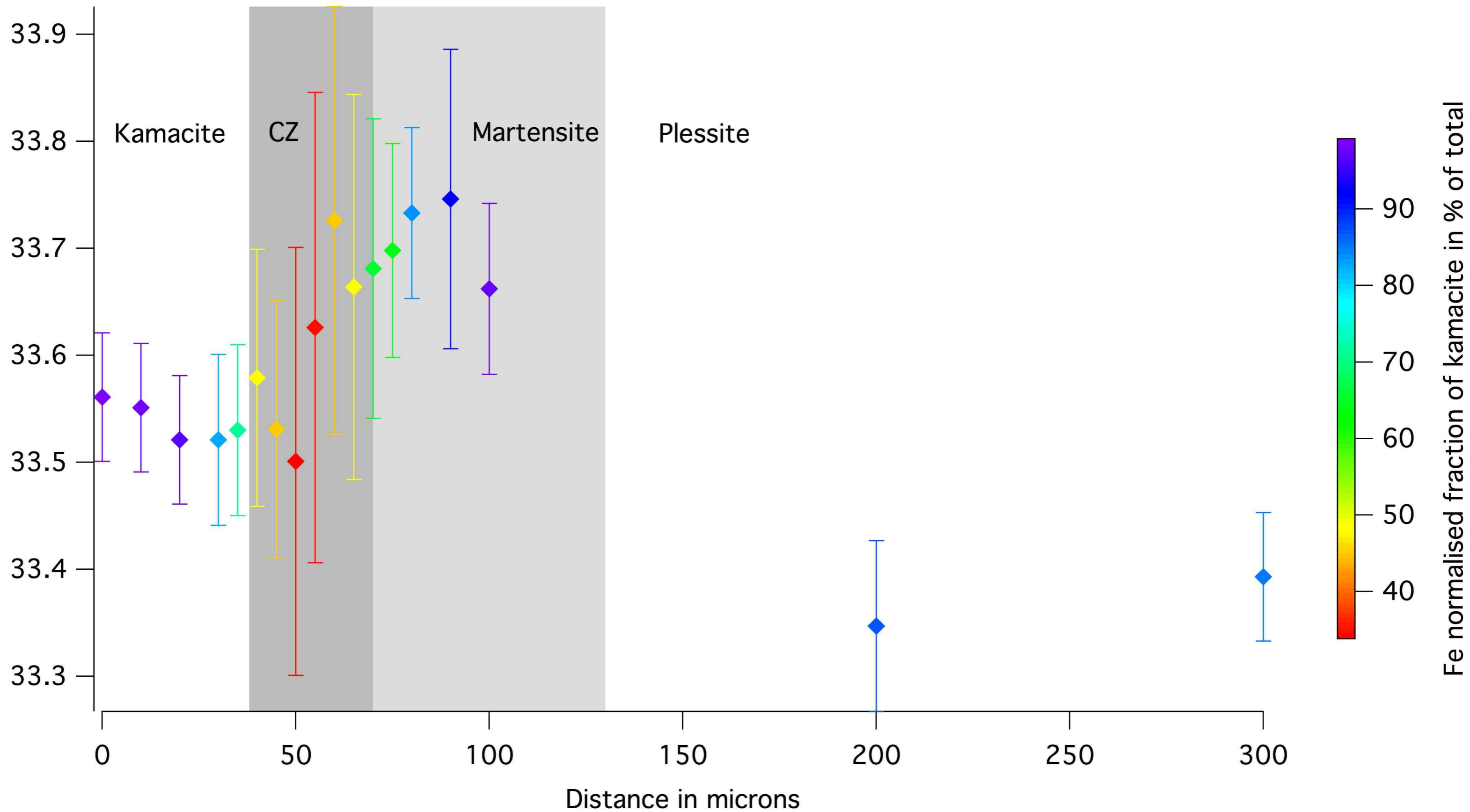

Supplement: Supplementary file 1 — Fig. S1: Variations in hyperfine fields of kamacite throughout the cloudy zone, from Mössbauer spectra acquired along Profile 1 of the Esquel meteorite. [file MAPS-52-925-s001.pdf]

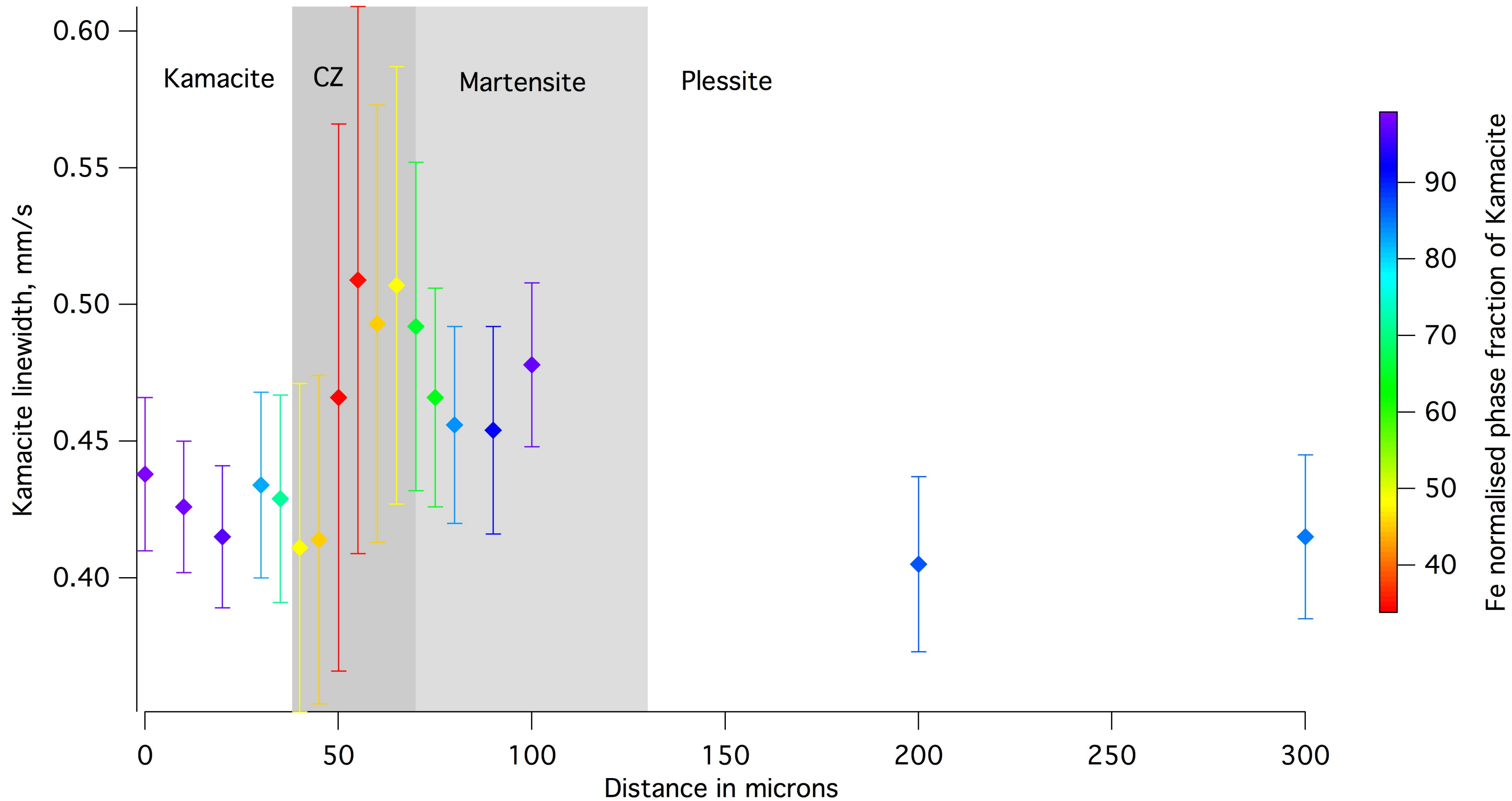

Supplement: Supplementary file 2 — Fig. S2: Variations in linewidth (FWHM) of kamacite throughout the cloudy zone, from kamacite to plessite, as determined from Mössbauer spectra acquired along Profile 1 of the Esquel meteorite. [file MAPS-52-925-s002.pdf]

Kamacite B field, T

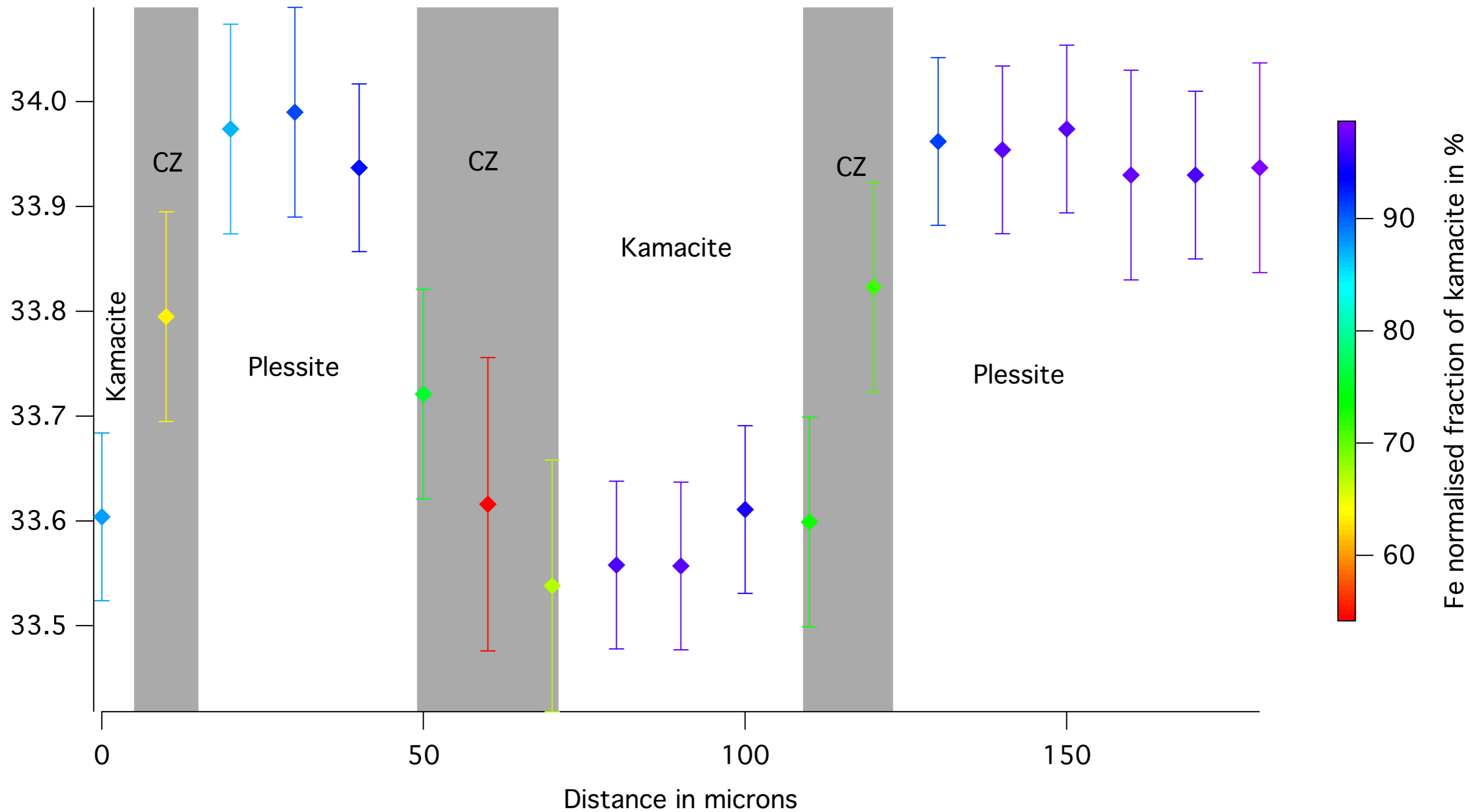

Supplement: Supplementary file 3 — Fig. S3: Variations in the hyperfine field of kamacite across the profile of the Tazewell meteorite. [file MAPS-52-925-s003.pdf]

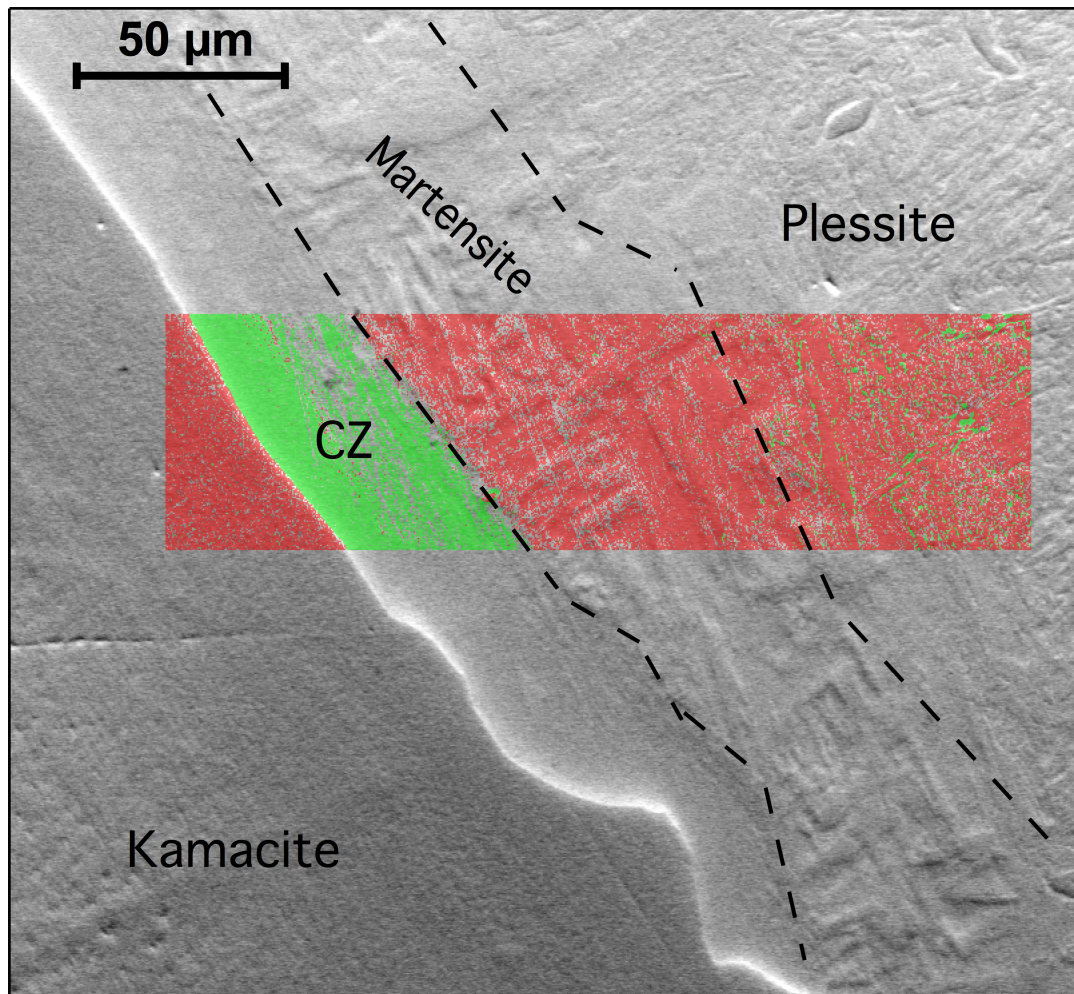

Supplement: Supplementary file 4 — Fig. S4: Overlay of phases observed by EBSD over a BSE image of the Esquel meteorite at a site where Profile 1 was taken. Bcc iron is shown in red; fcc Fe‐Ni phases are green. [file MAPS-52-925-s004.pdf]

80  $\mu\text{m}$

Kamacite

Martensite

Plessite

Schreibersite

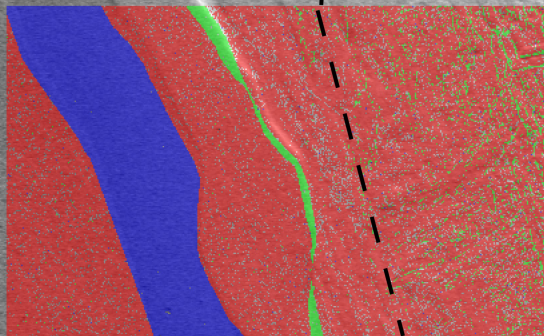

Supplement: Supplementary file 5 — Fig. S5: Overlay of phases observed by EBSD over a BSE image of the Esquel meteorite at a site where Profile 2 was taken. Bcc Fe (kamacite) is shown in red; fcc Fe‐Ni phases are green; schreibersite is blue. Some misalignment of images, due to beam drift, can be seen at the top of the EBSD image. [file MAPS-52-925-s005.pdf]

40  $\mu\text{m}$

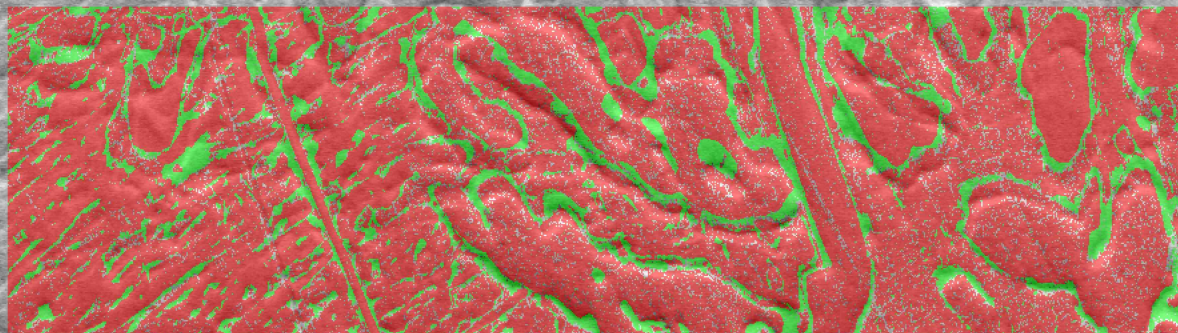

Supplement: Supplementary file 6 — Fig. S6: Overlay of phases observed by EBSD over a BSE image of the Esquel meteorite in plessite at the approximate location of the last spectrum in Profile 1. Bcc iron is shown in red; fcc Fe‐Ni phases are green. [file MAPS-52-925-s006.pdf]

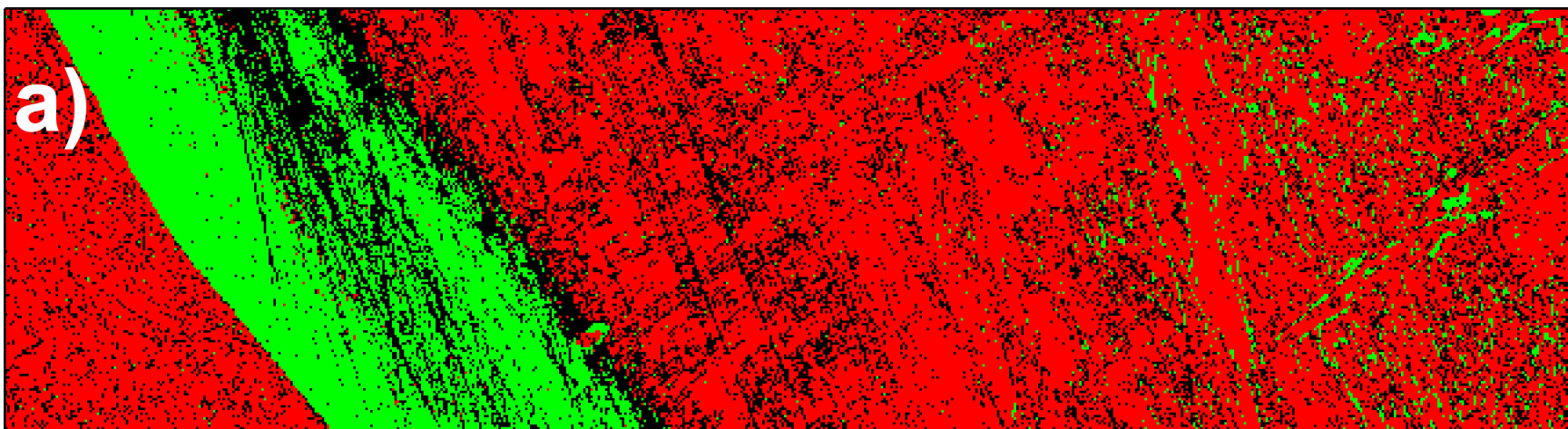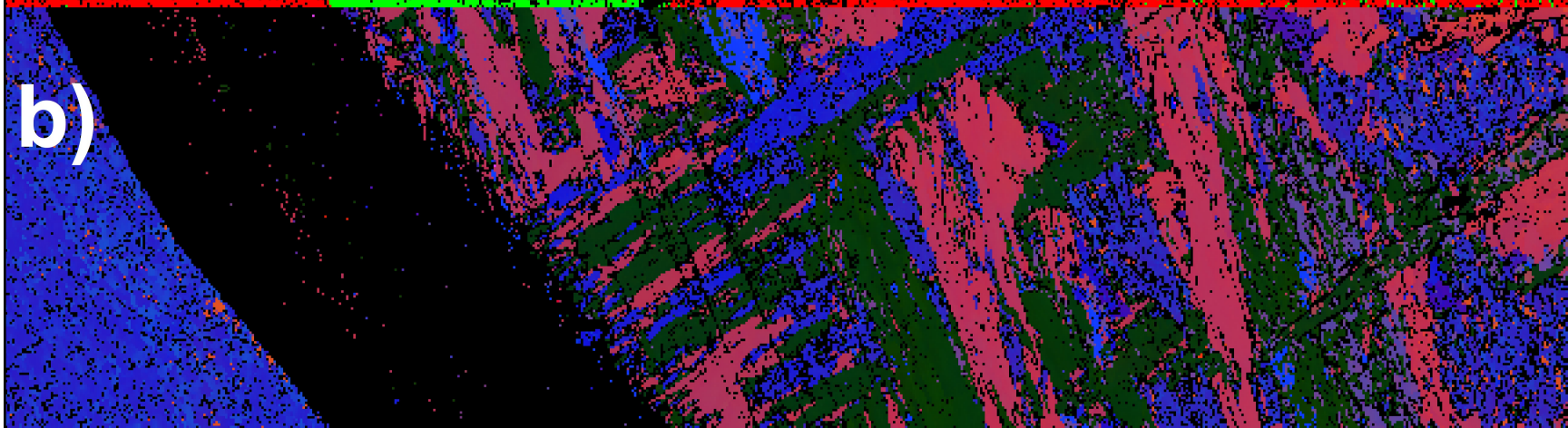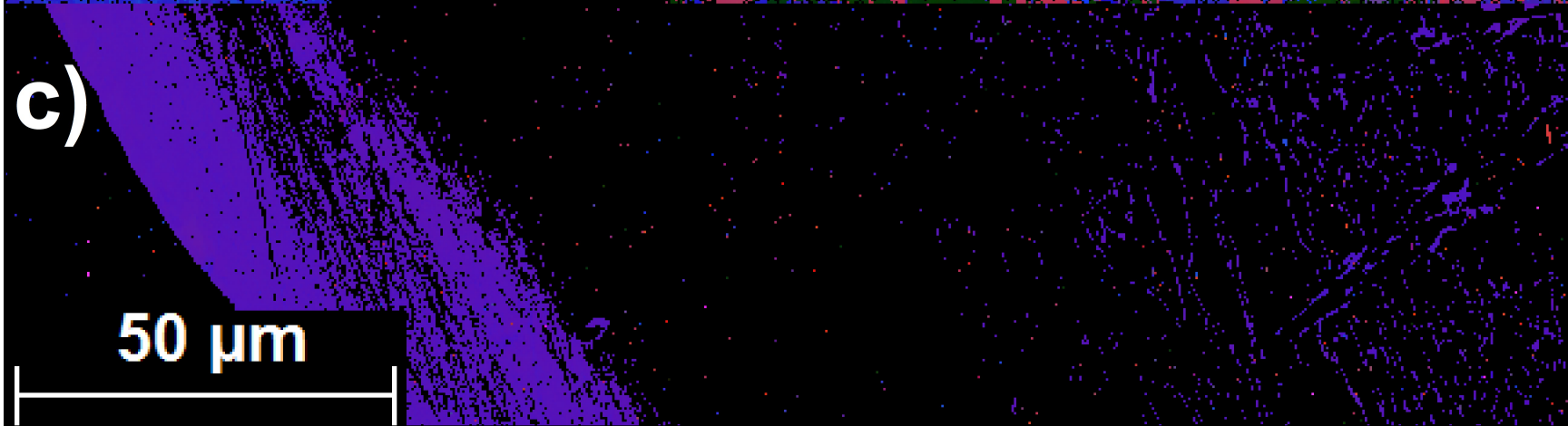

Supplement: Supplementary file 8 — Fig. S8: EBSD images of the area shown in Fig. S4 showing: a) phases observed; bcc is red, fcc is green. b) Euler angles of bcc grains. c) Euler angles of fcc grains (CZ and rims in plessite). For color explanation of Euler angles see legend in Fig. S10. [file MAPS-52-925-s008.pdf]

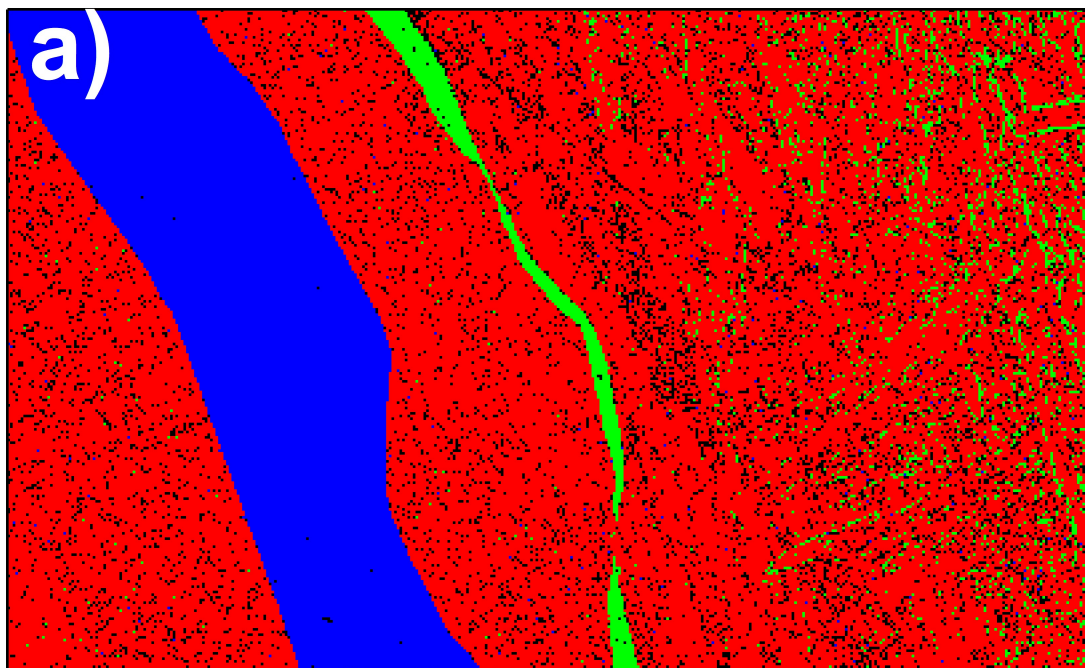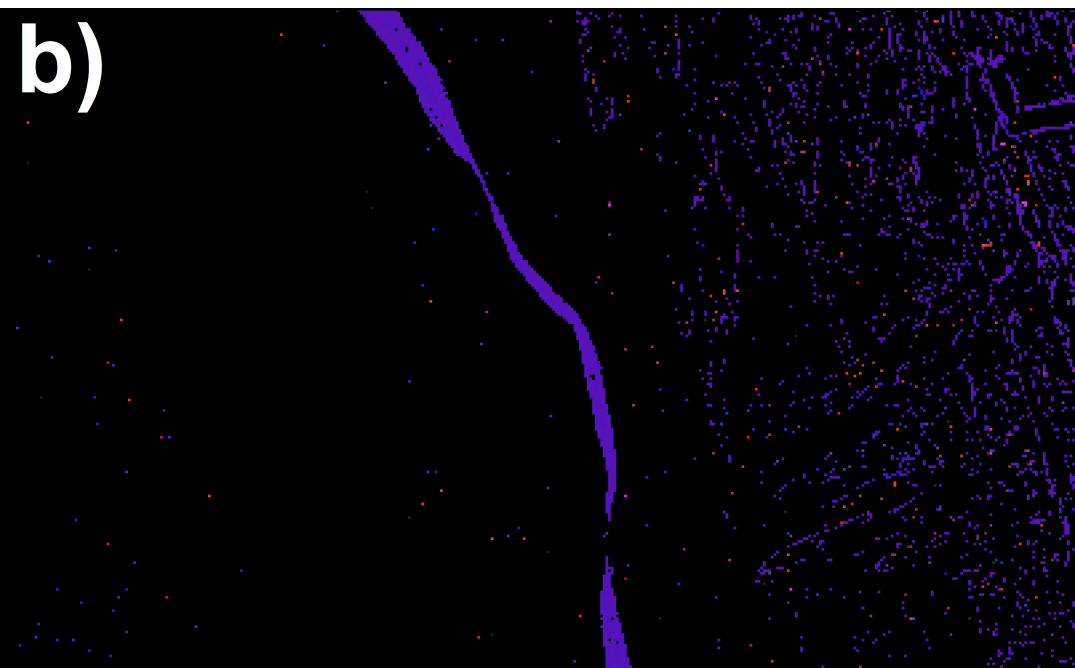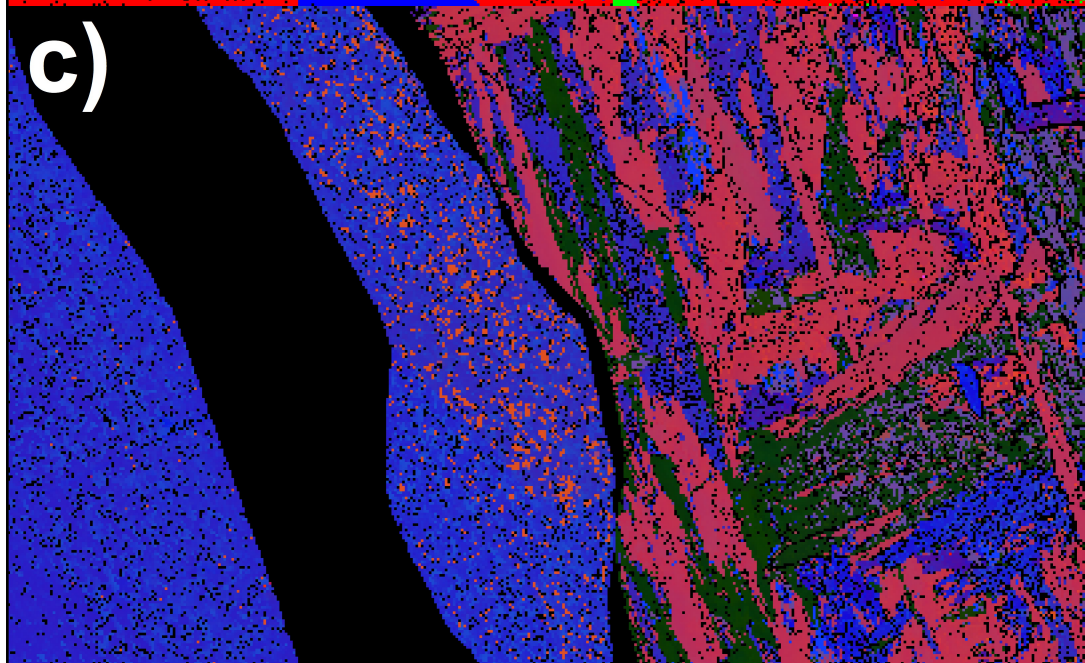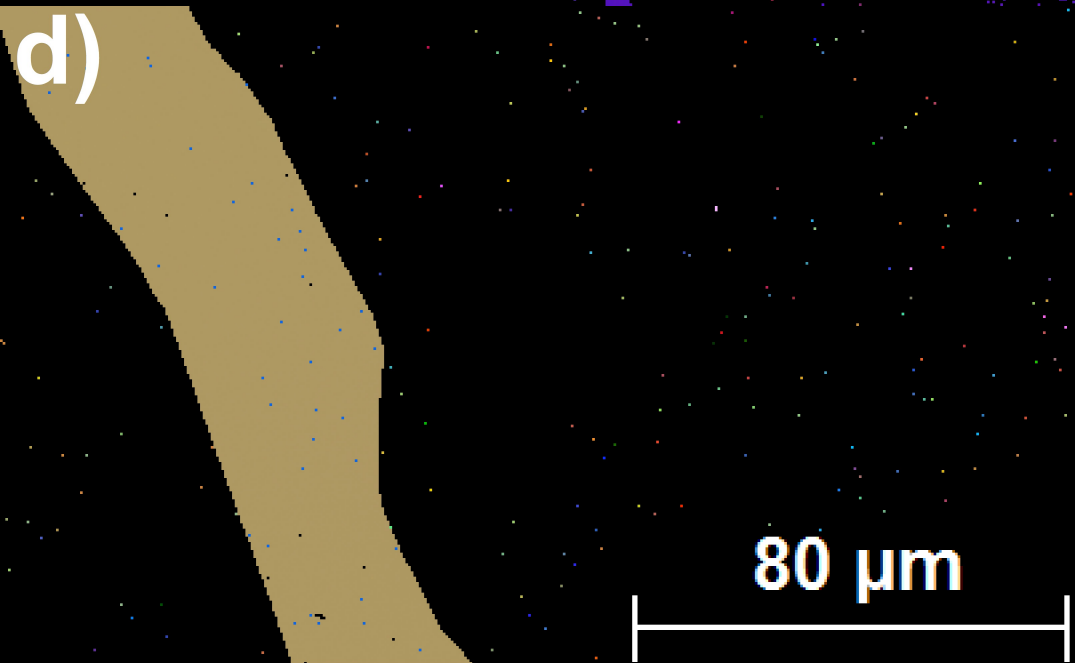

Supplement: Supplementary file 9 — Fig. S9: EBSD images of the area shown in Fig. S5 showing: a) phases observed; bcc is red, fcc is green, schreibersite is blue. b) Euler angles of fcc grains. c) Euler angles of bcc grains. d) Euler angle of schreibersite grain. Small single‐pixel sized, randomly oriented schreibersite grains are an artifact providing a qualitative indication of the number of misidentified and wrongly assigned pixels. For color explanation of Euler angles see legend in Fig. S10. [file MAPS-52-925-s009.pdf]
