# Supplementary material for: A high spatial resolution synchrotron Mössbauer study of the Tazewell IIICD and Esquel pallasite meteorites
Source: Meteorit Planet Sci. 2017 Mar 15;52(5):925–36. doi: 10.1111/maps.12841 (PMC5488627; doi:10.1111/maps.12841)
Supplement: Supplementary file 7 — Fig. S7: Pole figures of bcc Fe (left) and fcc Fe‐Ni (right) as obtained from plessite in the Esquel sample. [file MAPS-52-925-s007.pdf]

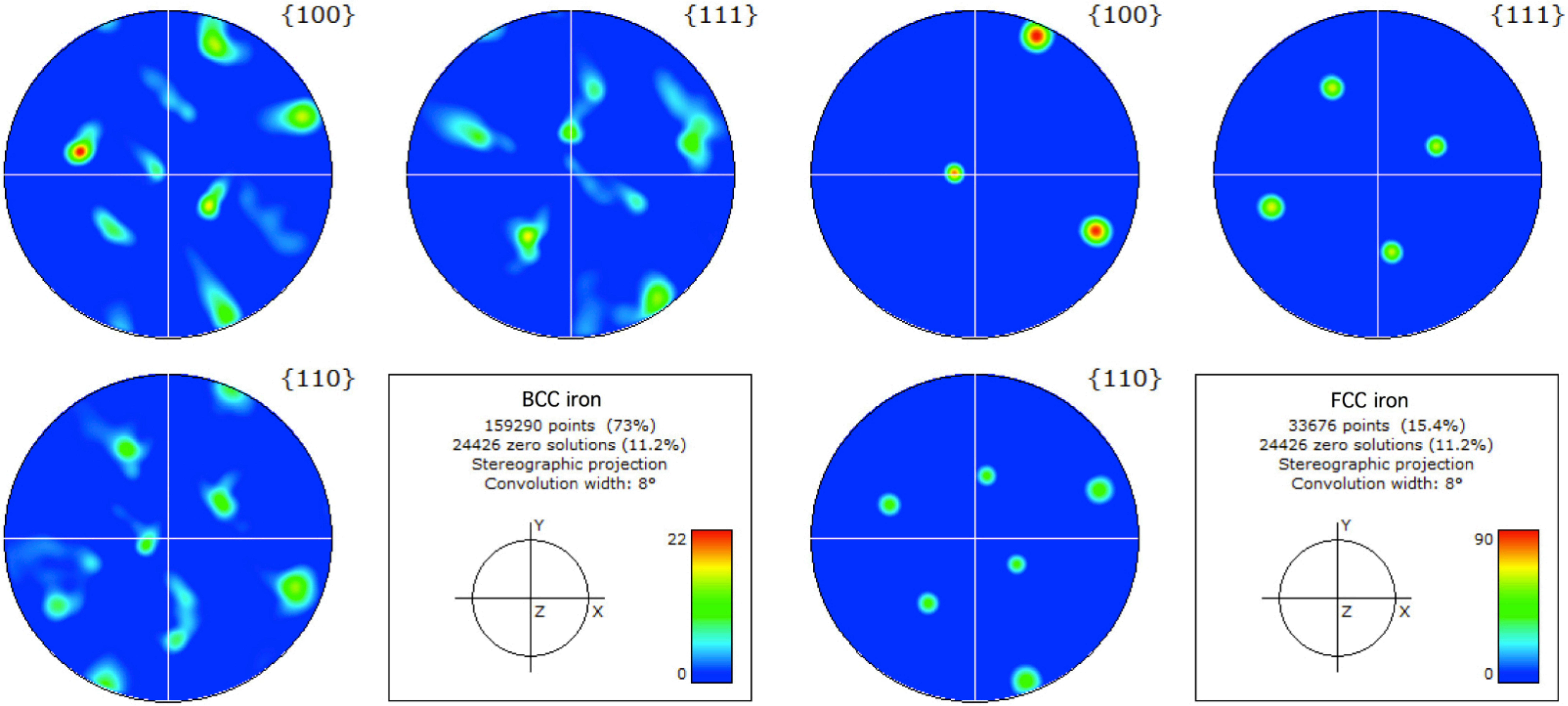
{100}
{111}
{100}
{111}
{110}
BCC iron
159290 points (73%)
24426 zero solutions (11.2%)
Stereographic projection
Convolution width: 8°
Y
Z
X
22
0
{110}
FCC iron
33676 points (15.4%)
24426 zero solutions (11.2%)
Stereographic projection
Convolution width: 8°
Y
Z
X
90
0
