# Supplementary material for: A high spatial resolution synchrotron Mössbauer study of the Tazewell IIICD and Esquel pallasite meteorites
Source: Meteorit Planet Sci. 2017 Mar 15;52(5):925–36. doi: 10.1111/maps.12841 (PMC5488627; doi:10.1111/maps.12841)
Supplement: Supplementary file 10 — Fig. S10: Legend for Euler plots. Color of a pixel is a combination from contributions from all three Euler angles. [file MAPS-52-925-s010.pdf]

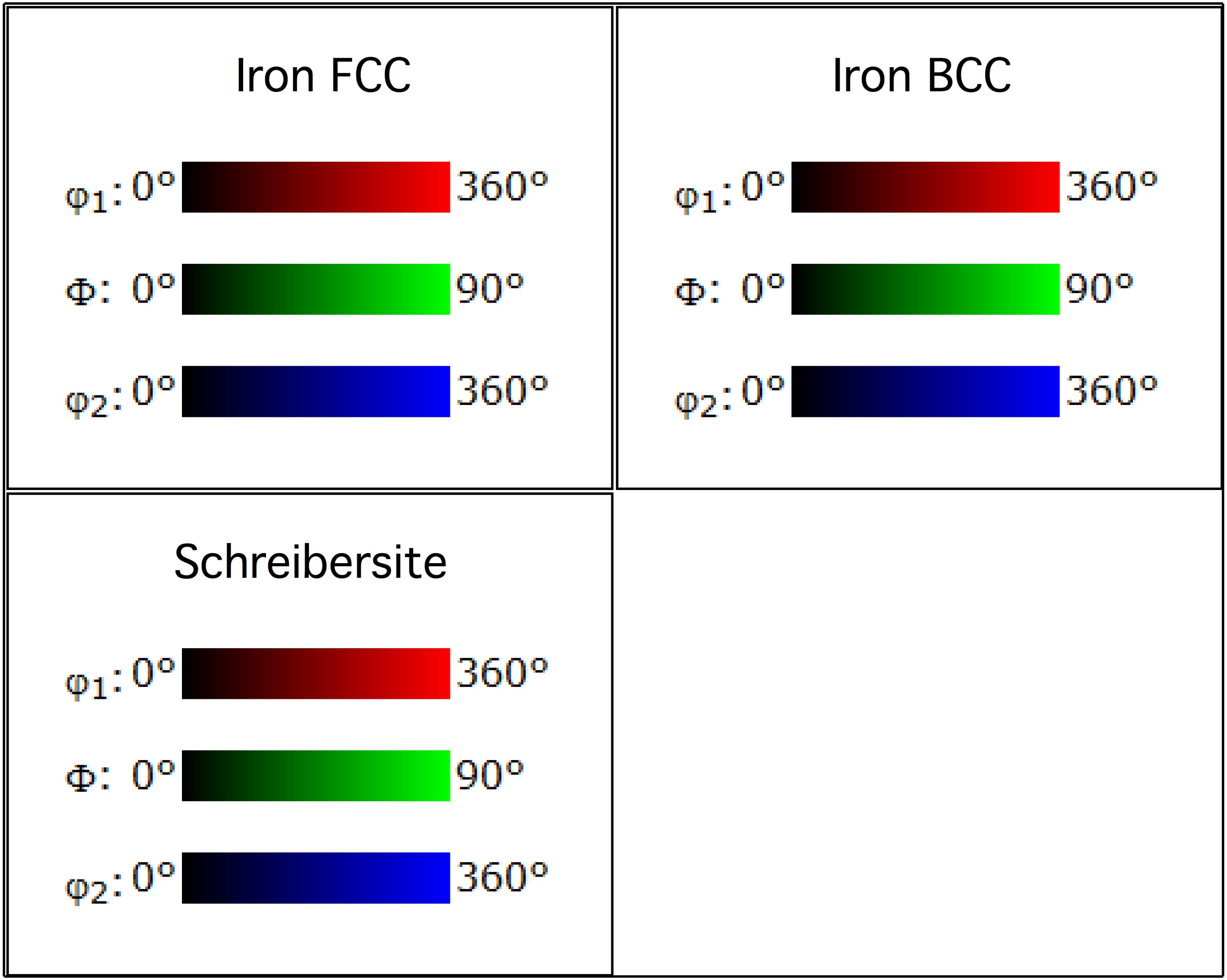
Iron FCC
φ1: 0° 360°
Φ: 0° 90°
φ2: 0° 360°
Iron BCC
φ1: 0° 360°
Φ: 0° 90°
φ2: 0° 360°
Schreibersite
φ1: 0° 360°
Φ: 0° 90°
φ2: 0° 360°
