# Supplementary material for: A high spatial resolution synchrotron Mössbauer study of the Tazewell IIICD and Esquel pallasite meteorites
Source: Meteorit Planet Sci. 2017 Mar 15;52(5):925–36. doi: 10.1111/maps.12841 (PMC5488627; doi:10.1111/maps.12841)
Supplement: Supplementary file 12 [file MAPS-52-925-s012.docx]

Supplemental Figure 1. Variations in hyperfine fields of kamacite throughout the cloudy zone, from Mössbauer spectra acquired Profile 1 of the Esquel meteorite.


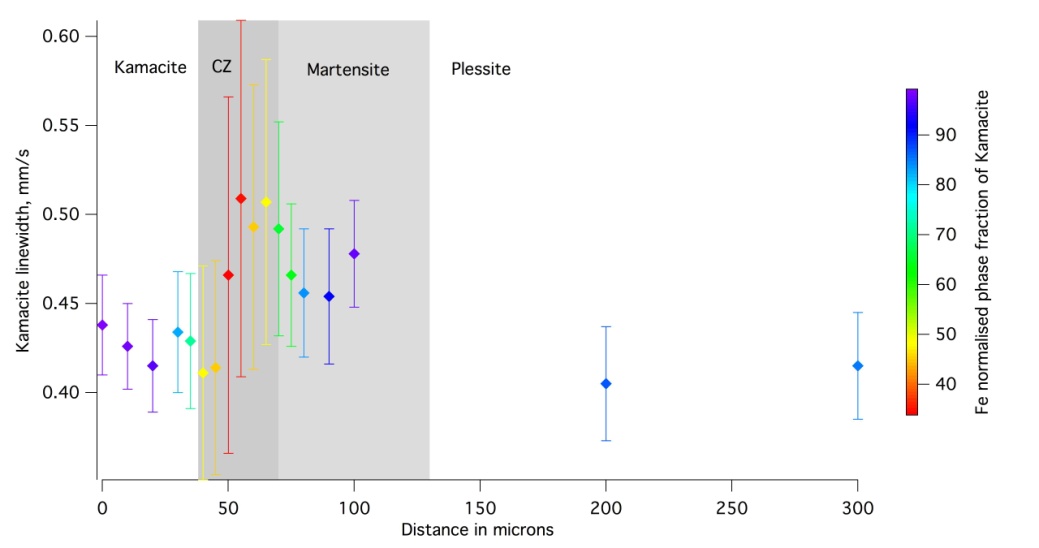


Supplemental Figure 2. Variations in linewidth (FWHM) of kamacite throughout the cloudy zone, from kamacite to plessite, as determined from Mössbauer spectra acquired along Profile 1 of the Esquel meteorite.


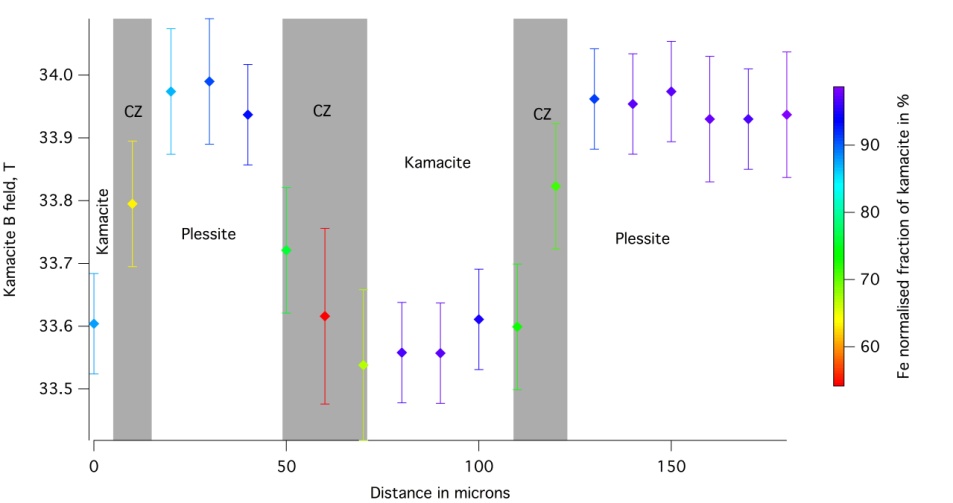


Supplemental Figure 3. Variations in the hyperfine field of kamacite across the profile of the Tazewell meteorite.


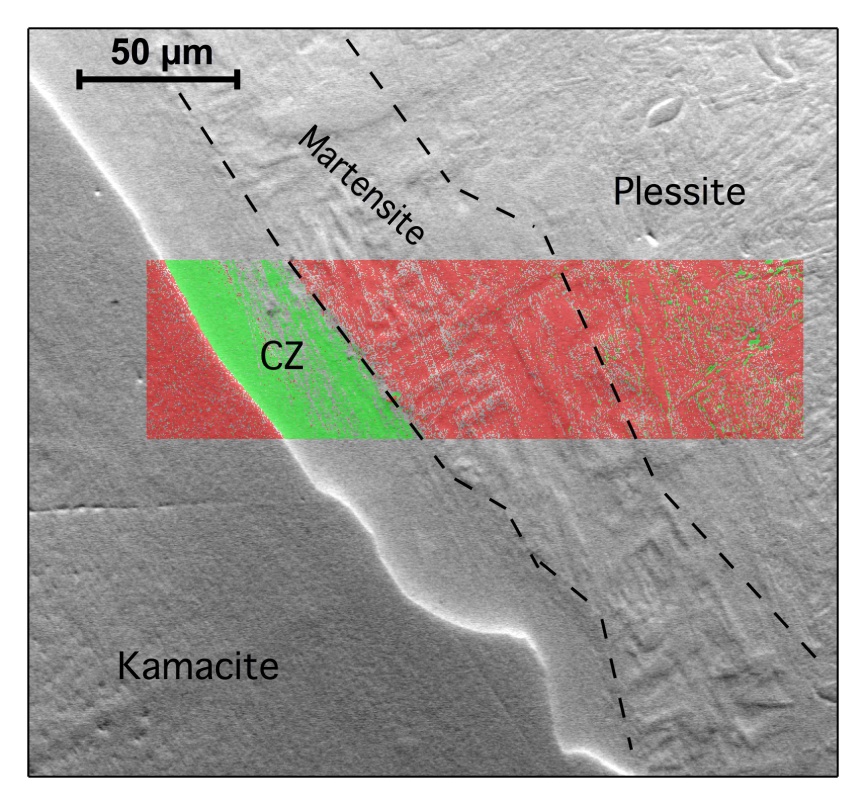


Supplemental Figure 4. Overlay of phases observed by EBSD over a BSE image of the Esquel meteorite at a site where Profile 1 was taken. Bcc iron is shown in red; fcc Fe-Ni phases are green.


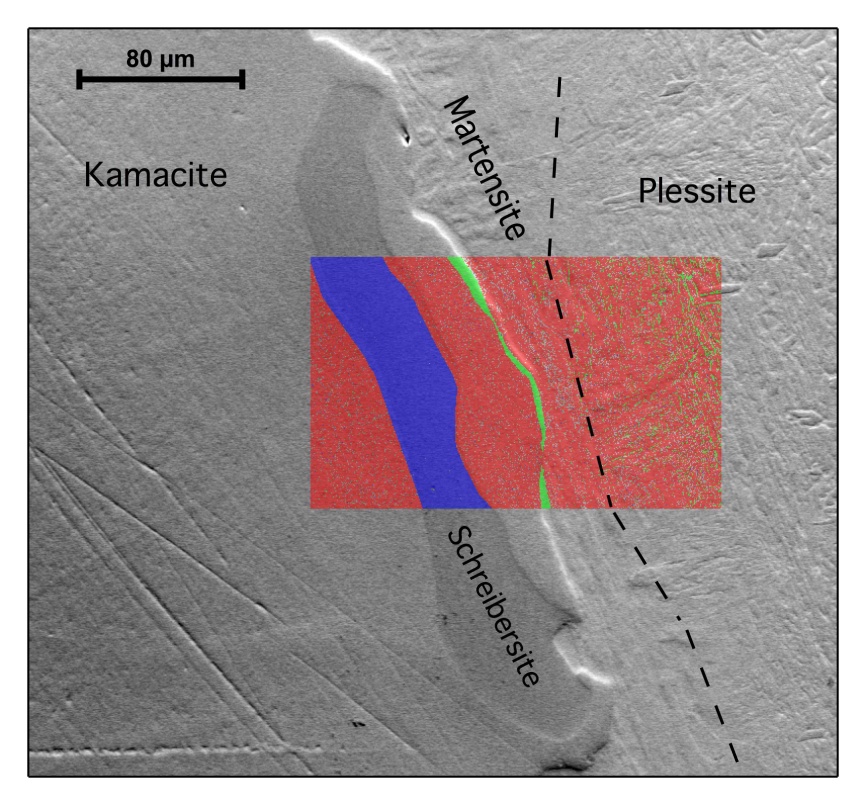


Supplemental Figure 5. Overlay of phases observed by EBSD over a BSE image of the Esquel meteorite at a site where Profile 2 was taken. Bcc Fe (kamacite) is shown in red; fcc Fe-Ni phases are green; schreibersite is blue. Some misalignment of images, due to beam drift, can be seen at the top of the EBSD image.


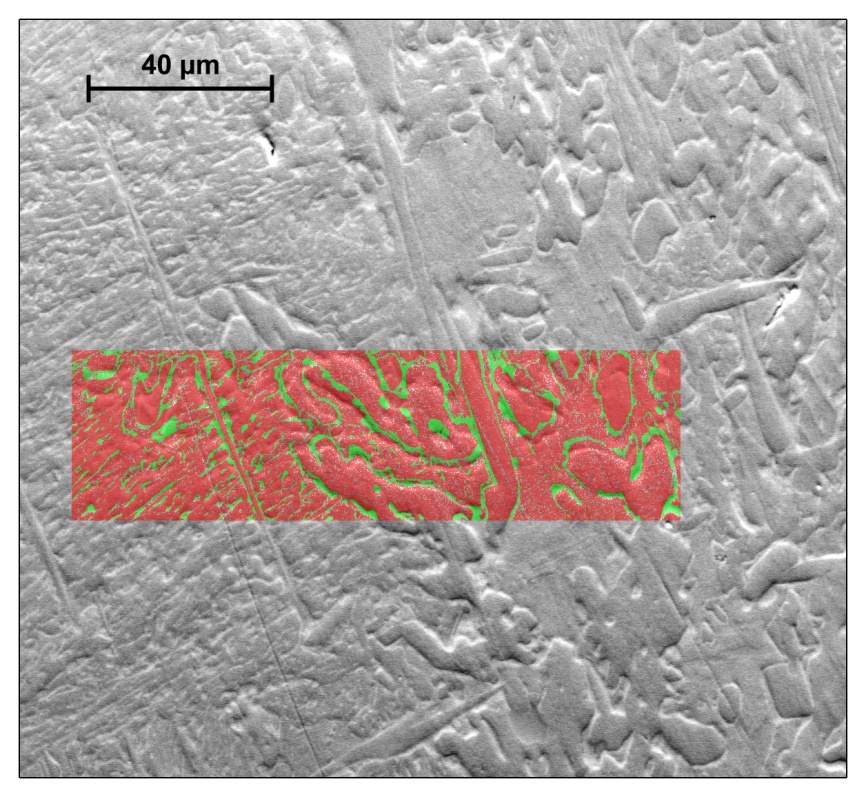


Supplemental Figure 6. Overlay of phases observed by EBSD over a BSE image of the Esquel meteorite in plessite at the approximate location of the last spectrum in Profile 1. Bcc iron is shown in red; fcc Fe-Ni phases are green.


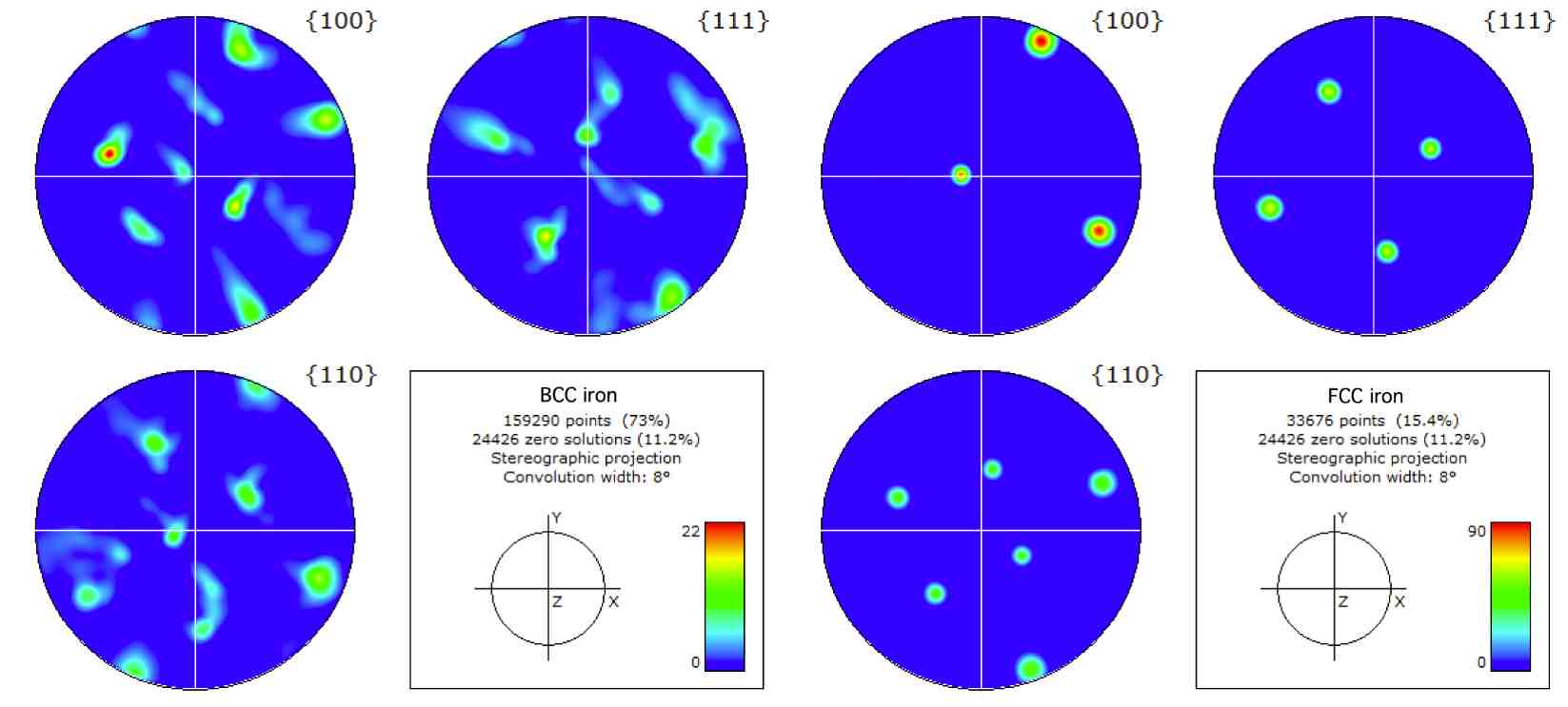


Supplemental Figure 7. Pole figures of bcc Fe (left) and fcc Fe-Ni (right) as obtained from plessite in the Esquel sample.


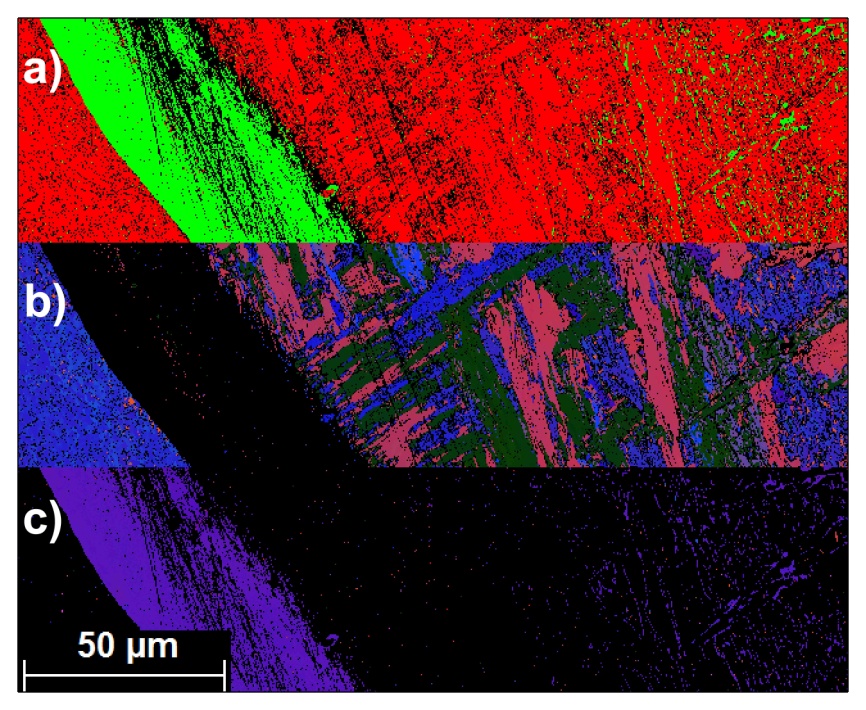


Supplemental Figure 8. EBSD images of the area shown in Supp. Fig. 4 showing: **a)** phases observed; bcc is red, fcc is green; **b)** Euler angles of bcc grains; **c)** Euler angles of fcc grains (CZ and rims in plessite). For color explanation of Euler angles see legend in Supplemental Figure 10.


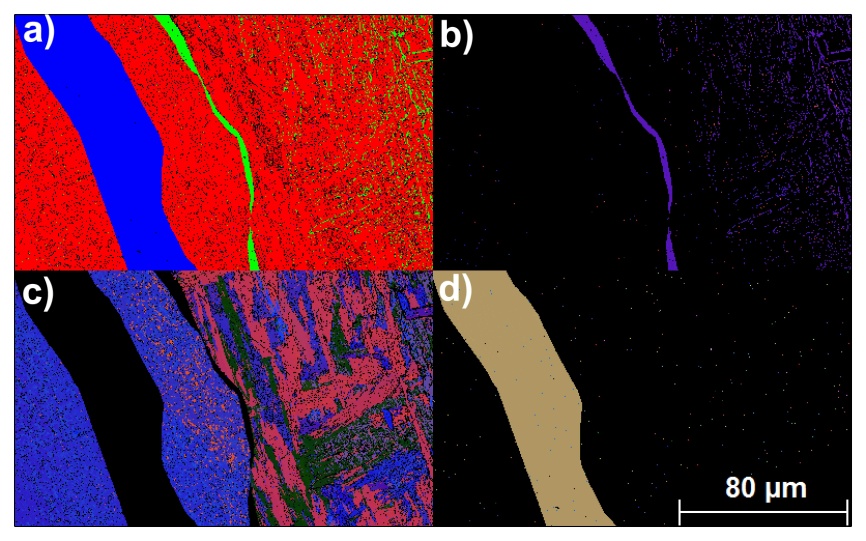


Supplemental Figure 9. EBSD images of the area shown in Supp. Fig. 5 showing: **a)** phases observed; bcc is red, fcc is green, schreibersite is blue; **b)** Euler angles of fcc grains; **c)** Euler angles of bcc grains; **d)** Euler angle of schreibersite grain. Small single-pixel sized, randomly oriented schreibersite grains are an artifact providing a qualitative indication of the number of misidentified and wrongly assigned pixels. For color explanation of Euler angles see legend in Supplemental Figure 10.


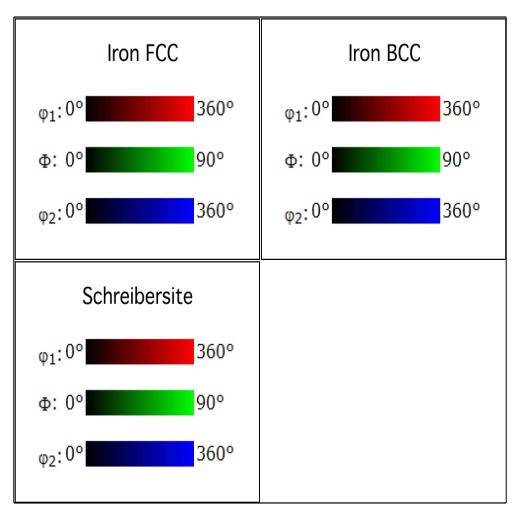


Supplemental Figure 10. Legend for Euler plots. Color of a pixel is a combination from contributions from all three Euler angles.
